# Insights into Platypus Population Structure and History from Whole-Genome Sequencing

**DOI:** 10.1093/molbev/msy041

**Published:** 2018-03-20

**Authors:** Hilary C Martin, Elizabeth M Batty, Julie Hussin, Portia Westall, Tasman Daish, Stephen Kolomyjec, Paolo Piazza, Rory Bowden, Margaret Hawkins, Tom Grant, Craig Moritz, Frank Grutzner, Jaime Gongora, Peter Donnelly

**Affiliations:** 1 Wellcome Centre for Human Genetics, University of Oxford, Oxford, United Kingdom; 2 Wellcome Trust Sanger Institute, Wellcome Genome Campus, Hinxton, United Kingdom; 3 Sydney School of Veterinary Science, The University of Sydney, Sydney, NSW, Australia; 4 Department of Genetics and Evolution, School of Biological Sciences, The University of Adelaide, Adelaide, SA, Australia; 5 School of Biological Sciences, Lake Superior State University, Sault Sainte Marie, MI; 6 Department of Medicine, Faculty of Medicine, Imperial College, London, United Kingdom; 7 Taronga Zoo, Mosman, NSW, Australia; 8 School of Biological, Earth and Environmental Sciences, University of New South Wales, Sydney, NSW, Australia; 9 Research School of Biology and Centre for Biodiversity Analysis, The Australian National University, Acton, ACT, Australia; 10 Department of Statistics, University of Oxford, Oxford, United Kingdom

**Keywords:** monotremes, population structure, genomics, evolution

## Abstract

The platypus is an egg-laying mammal which, alongside the echidna, occupies a unique place in the mammalian phylogenetic tree. Despite widespread interest in its unusual biology, little is known about its population structure or recent evolutionary history. To provide new insights into the dispersal and demographic history of this iconic species, we sequenced the genomes of 57 platypuses from across the whole species range in eastern mainland Australia and Tasmania. Using a highly improved reference genome, we called over 6.7 M SNPs, providing an informative genetic data set for population analyses. Our results show very strong population structure in the platypus, with our sampling locations corresponding to discrete groupings between which there is no evidence for recent gene flow. Genome-wide data allowed us to establish that 28 of the 57 sampled individuals had at least a third-degree relative among other samples from the same river, often taken at different times. Taking advantage of a sampled family quartet, we estimated the de novo mutation rate in the platypus at 7.0 × 10^−9^/bp/generation (95% CI 4.1 × 10^−9^–1.2 × 10^−8^/bp/generation). We estimated effective population sizes of ancestral populations and haplotype sharing between current groupings, and found evidence for bottlenecks and long-term population decline in multiple regions, and early divergence between populations in different regions. This study demonstrates the power of whole-genome sequencing for studying natural populations of an evolutionarily important species.

## Introduction

Next-generation sequencing technologies have greatly facilitated studies into the diversity and population structure of nonmodel organisms. For example, whole-genome sequencing (WGS) has been applied to investigate demographic history and levels of inbreeding in primates, with implications for conservation (Locke et al. 2011; Prado-Martinez et al. 2013; McManus et al. 2015; Xue et al. 2015; Abascal et al. 2016). It has also been used to study domesticated species such as pigs (Bosse et al. 2014), dogs (Freedman et al. 2014), maize (Hufford et al. 2012), and bees (Wallberg et al. 2014), to infer the origins of domestication, its effect on effective population size (*N*_e_) and nucleotide diversity, and to identify genes under selection during this process. Some studies have identified signatures of introgression (Bosse et al. 2014) or admixture (Miller et al. 2012; Lamichhaney et al. 2015) between species, which is important to inform inference of past *N*_e_. Others have used WGS data to identify particular genomic regions contributing to evolutionarily important traits, such as beak shape in Darwin’s finches (Lamichhaney et al. 2015), mate choice in cichlid fish (Malinsky et al. 2015), and migratory behavior in butterflies (Zhan et al. 2014). Here, we describe a population resequencing study of the platypus (*Ornithorhynchus anatinus*), which is one of the largest such studies of nonhuman mammals, and the first for a nonplacental mammal.

In addition to laying eggs, platypuses have a unique set of characteristics (Grant 2007), including webbed feet, a venomous spur (only in males), and a large bill that contains electroreceptors used for sensing their prey. Their karyotype is 2*n* = 52 (Bick and Sharman 1975), and they have five different male-specific chromosomes (named Y chromosomes), and five different chromosomes present in one copy in males and two copies in females (X chromosomes), which form a multivalent chain in male meiosis (Grützner et al. 2004).

Though apparently secure across much of its eastern Australian range, the platypus has the highest conservation priority ranking among mammals when considering phylogenetic distinctiveness (Isaac et al. 2007). Given concerns about the impact of climate change (Klamt et al. 2011), disease (Gust et al. 2009) and other factors on platypus populations, there is a need to better understand past responses of platypus populations to climate change, and the extent of connectivity across the species range.

**Fig. 1.**
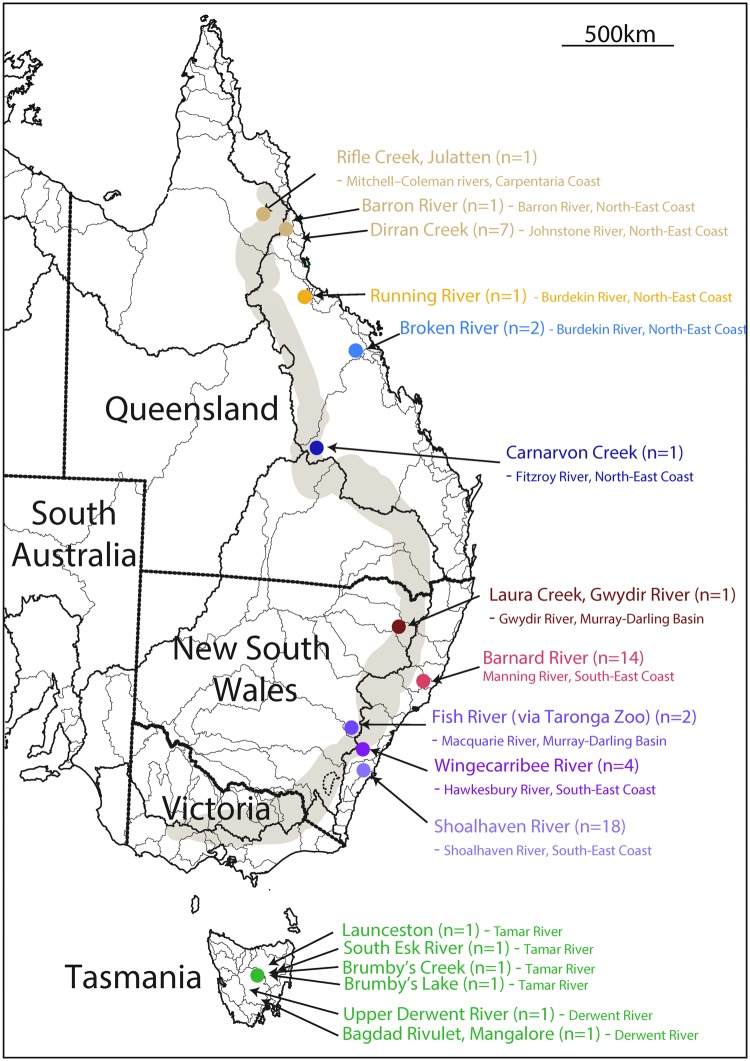
Map showing our sampling locations within river regions in eastern and southeastern Australia. The lighter lines on the figure are rivers and the darker lines demarcate catchment areas. The waterways our samples come from are indicated by arrows, with sample sizes in brackets after the river name. The specific catchments these rivers fall into and their corresponding larger drainage division are indicated in small letters after the river name. The text colors correspond to those used in later figures. The transparent gray region represents the Great Dividing Range (GDR). Note that all samples except those from the Fish River, Gwydir River, and Rifle Creek come from river basins that drain east from the GDR. This map is adapted from one obtained from the Australian Bureau of Meteorology (www.bom.gov.au/water/geofabric/documents/BOM002_Map_Poster_A3_Web.pdf) under a CC-BY license (https://creativecommons.org/licenses/by/2.0/).

The first platypus genome assembly (ornAna1) was generated using established whole-genome shotgun methods (Warren et al. 2008) from a female from the Barnard River in New South Wales (NSW) (see fig. 1). This assembly was highly fragmented and did not contain any sequence from the Y chromosomes. The initial genome paper included only a limited analysis of interindividual variation and population structure based on 57 polymorphic retrotransposon loci. Subsequently, several other studies have investigated diversity and population structure using microsatellites or mitochondrial DNA (mtDNA) (Kolomyjec et al. 2009, 2013; Gongora et al. 2012; Furlan et al. 2013) (table 1). They reported much stronger differences between rather than within river systems, but found some evidence of migration between rivers that were close together, implying limited overland dispersal.

**Table 1.** Summary of Genetic Data and Samples Used in This and Previous Studies.

Paper	Number of Samples	Sampling Locations	Genetic Data
Warren et al. (2008)	90	QLD, NSW, VIC, TAS, South Australia	57 retrotransposons
Kolomyjec et al. (2009)	120	Five river systems in NSW, predominantly Hawkesbury and Shoalhaven	12 microsatellites
Gongora et al. (2012)	284	22 river systems across whole platypus range	Haplotypes of mitochondrial control region and cytochrome b gene
Furlan et al. (2013)	752	33 river systems across NSW, Victoria, TAS	Three microsatellites, two mitochondrial haplotypes
This study	57	12 river systems across QLD, NSW, TAS	6.7 million SNPs from WGS data

Each locus has a genealogy, which can be correlated for loci near each other on the same chromosome. At a particular locus, the actual genealogy is the result of chance events in the history of the sample, with the probabilities involved being affected by the demographic history of the population. Thus, even perfect genealogies from a single locus (such as the mitochondrial control region; Gongora et al. 2012) contain limited information about the demographic history of the population or about the ancestral relationships among different populations (Novembre and Ramachandran 2011). Incomplete information from several loci, such as microsatellites (Furlan et al. 2013), also contains limited information for the same reasons. We thus chose to sequence entire genomes of the study individuals to give the widest possible source or information about their ancestral history.

We sequenced the genomes of 57 platypuses from Queensland (QLD), New South Wales (NSW), and Tasmania (TAS) (fig. 1; supplementary tables S1 and S2, Supplementary Material online), in order to gain insights into the population genetics of the species. We investigated the differentiation between subpopulations, the relative historical population sizes and structure, and the extent of relatedness between the individuals sampled, which could be informative about the extent of individual platypus dispersal.

## Results

### Genome Reassembly and SNP Calling

We sequenced 57 platypus samples at 12–21× coverage, one in duplicate. We used the improved genome assembly ornAna3 for all analyses, and ran standard software to jointly call variants across all samples (PLATYPUS; Rimmer et al. 2014). The variant calls were filtered to produce a set of 6.7 M stringently filtered SNPs across 54 autosomal scaffolds comprising 965 Mb of the assembly. Although the sequence coverage was variable across samples, we found that the number of variants called was not affected by the sequence coverage for the individual (supplementary fig. S1, Supplementary Material online).

### Data Quality

We undertook two different approaches to assess the quality of our SNP callset. In the first approach, two separate DNA samples from a single individual were sequenced. These were processed in identical fashion to all the other sequence data, with the processing blind to the fact that they were duplicates. After processing and SNP calling, we then compared the genotypes in the two samples from this individual. The rate of discordant genotypes between the two duplicate samples was very low (2.20 × 10^−^^3^ per SNP; 1.62 × 10^−^^5^/bp; supplementary table S3, Supplementary Material online); because an error in either duplicate could lead to discordant genotypes, this would lead to an estimated error rate of 1.10 × 10^−^^3^ per SNP and 8.1 × 10^−^^6^/bp. During our analyses, we also discovered we had sampled a family quartet of two parents and two offspring (supplementary table S4, Supplementary Material online). This allowed us to use a second approach to assess the quality of our SNP callset by using the rate of Mendelian errors. We found a Mendelian error rate of 1.10 × 10^−^^3^ per SNP. In some configurations, an error in any one of the four genotypes would result in a Mendelian error; in others, an error would not be detected. Both approaches suggest an error rate of order 0.001 per genotype, suggesting that the data set used in the analyses is of high quality. Our genotype error rate compares favorably with previous work in mountain gorillas (Xue et al. 2015) despite our lower sequencing coverage.

Sequencing a quartet also allowed us to estimate the switch error rate of haplotypes inferred in the new reference genome compared with the original assembly. Switch errors are changes in the pattern of inheritance in the two offspring, either due to errors or real recombination events (Browning and Browning 2011). We found that the switch error rate was reduced by nearly 80% for ornAna3 compared with ornAna1 (supplementary section S1 and table S5, Supplementary Material online), indicating the new assembly contains far fewer errors. We conclude that the improved reference genome and stringent filters we have used mean that our data set is of high quality.

### Relatives

Unlike earlier studies, our genome-scale data allowed us to look for relatives among our samples. Using the KING algorithm (Manichaikul et al. 2010) as implemented in VCFtools (Danecek et al. 2011), we identified many pairs of relatives (supplementary table S4, Supplementary Material online). In addition to a first-degree relative pair, we had intentionally sequenced (a father–daughter pair from Taronga Zoo) we found we had sampled the aforementioned quartet from the Shoalhaven River, as well as the quartet mother’s sister. Additionally, there were 26 pairs of second- or third-degree relatives, in all cases from the same river or creek, or closely connected waterways, involving 28 of our 57 samples. For the analyses in this paper, except where otherwise noted, we used a set of 43 unrelated samples, indicated in supplementary table S1, Supplementary Material online.

### De Novo Mutation Rate

We identified putative de novo mutations in the two offspring in the family quartet using the Bayesian filter incorporated into PLATYPUS, and further filtered them to remove any putative de novo mutation which was seen in any other sample. This gave us a total of 12 de novo mutations in the quartet, 6 in each offspring, and we estimated the de novo mutation rate at 7 × 10^−^^9^/bp/generation (95% CI 4.1 × 10^−^^9^–1.2 × 10^−^^8^/bp/generation). See supplementary section S3, Supplementary Material online, for more details.

### Dispersion and Inbreeding

Of the quartet individuals, the mother, her sister, and her two offspring were sampled in a pool at a junction ∼2 km downstream of the point where the father was found, which was in Jerrabattgulla Creek, a small tributary of the Shoalhaven River. Although the male offspring was first captured as a juvenile, because of difficulties in determining the age of adult platypuses, it is not possible to tell whether the two offspring were born as dizygotic twins or at different times, which would indicate that their parents mated in >1 year.

Given that we found so many instances of close relative pairs within the same river, it was natural to ask whether inbreeding is common in platypus. We examined long runs of homozygosity (LROHs) to investigate levels of inbreeding in the samples. Supplementary figure S2, Supplementary Material online, shows *F*_ROH_, the estimated fraction of the analyzed genome that is in LROHs (see Materials and Methods). The Carnarvon sample stands out, with *F*_ROH_ estimated at 24.4%, but several other samples have *F*_ROH_ higher than 10% (N745 and N711 from NQLD, N730 from the Gwydir River, N746 from the Broken River, N724 from the Barnard River, and N710 from Tasmania).

The length of homozygous segments depends on the recombination rate and number of generations since the most recent common ancestor of the two haplotypes. Since we do not know the fine-scale recombination rate in platypus, and estimation of segment length is complicated by the fragmented nature of the assembly, which may lead to ROHs being truncated artificially, for example, by scaffold ends (supplementary fig. S3, Supplementary Material online), interpretation of the observed distribution of segment lengths is challenging. However, supplementary figure S4, Supplementary Material online, shows that the samples clearly fall into two groups: the north QLD (NQLD), central QLD (CQLD), and Gwydir samples (group 1) all have more ROHs than the other NSW and Tasmanian samples (group 2), but these have lower mean length than in some of the group 2 samples, and both groups contain samples with high overall *F*_ROH_. This undoubtedly reflects differences in demographic history. In the case of the Carnarvon sample (N753), it is difficult to disentangle true inbreeding from low historical *N*_e_, since we only have one sample from this location, but the overall *F*_ROH_ could be consistent with a mating between first-degree relatives. However, since N724 appears to be an outlier among the Barnard River samples, it may be that this individual is derived from a mating of individuals as closely related as second-degree. Thus, we cannot rule out the possibility of close inbreeding in wild platypus populations.

### Population Structure

**Fig. 2.**
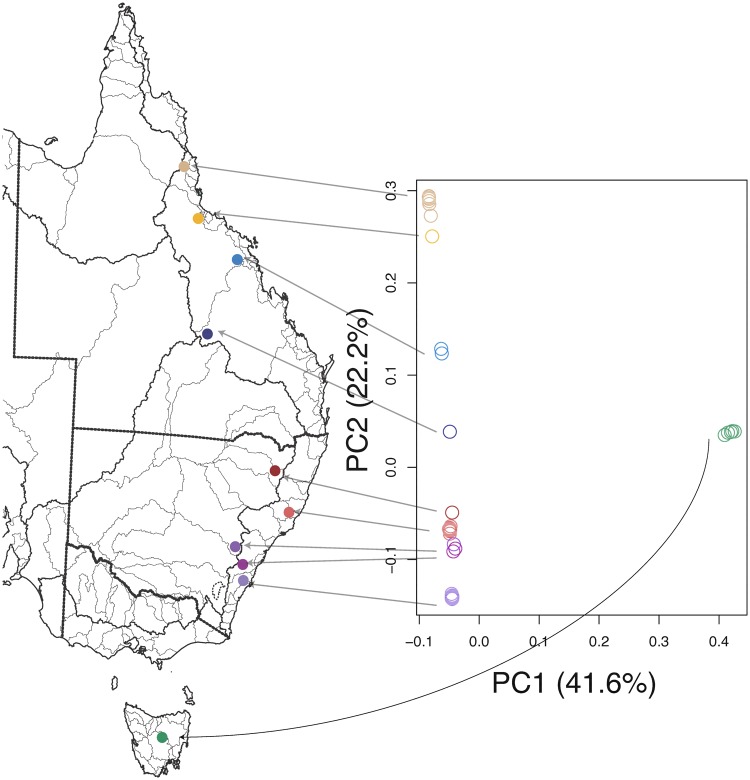
Principal component analysis on 43 unrelated samples. The first two principal components (PC1 and PC2) are shown, with the proportion of variance accounted for by each indicated in parentheses. Each unfilled circle in the plot represents an individual, with the colors of the circles corresponding to the sampling location. Circles are plotted at the value of the first two principal components for that individual.

We first ran a Principal Component Analysis (PCA) to summarize the genetic variation, using the stringent SNP set after filtering based on minor allele frequency and missingness. Figure 2 shows the first two principal components (PCs). The first PC separates the Tasmanian from the mainland samples and accounts for 41.6% of the variation, and the second separates the mainland samples on a north–south axis and accounts for 22.2% of the variation. If we prune the SNPs based on linkage disequilibrium (LD), the first two PCs are exchanged and account for 27.6% and 23.7% of the variation (data not shown).

In order to explore differences between regions, we divided the samples into groups based on the PCA results and the samples’ known geographical proximity to one another (see supplementary table S1, Supplementary Material online). The Barnard River group has 11 individuals, and is combined with the Gwydir River individual to form the north NSW group. The Shoalhaven River group has 12 individuals, and is combined with 2 individuals from the Wingecarribee River and one individual from the Fish River to form the central NSW group. Similarly, we grouped samples within each of Tasmania (*N* = 5) and NQLD (*N* = 7). The three samples from central QLD form their own group based on PCA and geography, and have been excluded from the following analyses due to low sample numbers.

The groups show different levels of nucleotide diversity, *π* (Nei and Li 1979) (average number of nucleotide differences between individuals per site), ranging from 4.73 × 10^−^^4^ in the north QLD samples to ∼1.02 × 10^−^^3^ in central NSW (CNSW) (table 2). A large proportion of the SNPs segregating in each region are only polymorphic in that region (supplementary fig. S5, Supplementary Material online): 34.5% of those in north QLD, 33.5% in north NSW (NNSW), 37.1% in central NSW and 72.1% in Tasmania (after downsampling to consider the same number of samples per region).

**Table 2.** Nucleotide Diversity (*π*) across Different Sampling Locations.

Sampling Location	No. of Samples	*π*
All	43	0.00117
Barnard	11	0.00100
Central NSW	15	0.00104
North NSW	12	0.00101
North QLD	7	0.00048
Shoalhaven	12	0.00100
TAS	5	0.00059

Note.—North NSW is Barnard + Gwydir, and Central NSW is Shoalhaven + Wingecarribee + Fish Rivers. Central QLD has only three samples and is excluded from this analysis.

There was high *F*_ST_ between the different regional groupings, with the highest value between Tasmania and north QLD (0.677) and the lowest between the Wingecarribee and Barnard groupings (0.077) (table 3). The *F*_ST_ values were slightly higher when we did not prune the SNPs based on local LD, since this unpruned SNP set retained many fixed differences between sampling locations (table 3). There were a large number of fixed differences between both the Tasmanian and north QLD samples and the reference individual (supplementary fig. S6, Supplementary Material online): 10% of the ∼6.7 million SNPs segregating in the 57 samples were fixed for the alternate allele in the five unrelated Tasmanian samples, and 7.3% were fixed in five randomly sampled unrelated north QLD samples.

**Table 3.** *F*_ST_ across Different Sampling Locations.

	Wingecarribee	North QLD	TAS	Barnard	Shoalhaven
Wingecarribee	–	0.364	0.544	0.086	0.088
North QLD	0.335	–	0.726	0.362	0.394
TAS	0.445	0.676	–	0.555	0.555
Barnard	0.078	0.335	0.459	–	0.145
Shoalhaven	0.078	0.361	0.463	0.126	–

Note.—The black numbers above the diagonal are calculated using SNPs before LD pruning, and the blue ones below the diagonal are calculated after LD pruning (see Materials and Methods).

**Fig. 3.**
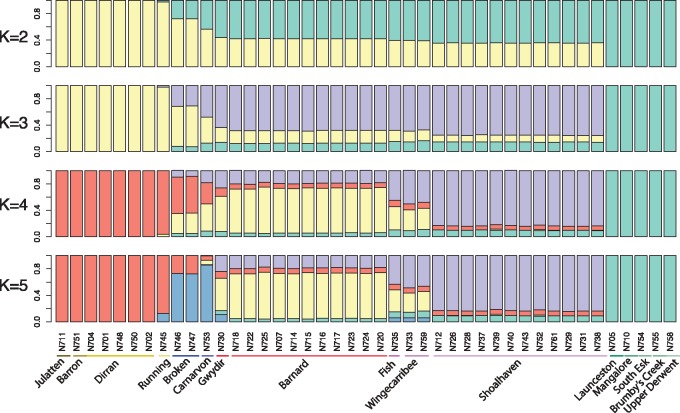
Population structure inferred from 43 unrelated individuals using STRUCTURE. Each individual is represented by a vertical bar partitioned into *K* colored segments that represent the individual’s estimated membership fractions in *K* clusters. Ten STRUCTURE runs at each *K* produced very similar results, and so the run with the highest likelihood is shown. The Broken River and Carnarvon samples are labeled as central QLD and the Running River sample as north QLD, even though these samples did not form part of large clusters on the PCA and were excluded from the groupings used in tables 2 and 3 and supplementary figures S5 and S6, Supplementary Material online.

We applied STRUCTURE (Pritchard et al. 2000), a Bayesian model-based clustering algorithm, to identify subgroupings within our sampling location groups and assign the samples to them without using any prior information. Figure 3 shows the results from the admixture model in STRUCTURE, in which individuals are allowed to have membership in more than one of the *K* subgroupings, or clusters. We emphasize that inference of membership in multiple clusters does not necessarily mean that an individual has recent admixture; rather, these clusters represent putative ancestral populations which may have contributed to modern-day populations.

For *K* = 2, the clusters are anchored by north QLD and Tasmania, with the central QLD and NSW groups inferred to be a mixture of these two. The next cluster at *K* = 3 corresponds to these central QLD and NSW individuals, and at *K* = 4, the north NSW (Barnard and Gwydir) individuals are delineated, along with individuals from the Fish and Wingecarribee Rivers. The additional cluster at *K* = 5 contributes the majority of the ancestry of the Carnarvon and Broken River samples, as well as a small amount of the ancestry of the samples from the Running River (from the Burdekin river system, like the Broken River individuals), and from the NSW rivers. When we ran STRUCTURE with *K* > 5, we found that the proportion of ancestry assigned to the additional clusters was always very low in all samples, and the posterior mean was close to 0 (i.e., the clusters were essentially empty), so, although slightly higher log likelihoods were observed for some runs at *K* = 8, we think that the simpler model with *K* = 5 represents the data better.

The most striking point in this figure is that the Wingecarribee and Fish River samples look more similar to the Gwydir and Barnard samples than they do to the Shoalhaven samples, despite being geographically adjacent to Shoalhaven (see fig. 1). On our PCA analyses, these samples fall in a position between the Shoalhaven and Barnard samples, but closer to the Barnard samples (rather than closer to Shoalhaven, as might be expected by their geographical location). We discuss possible explanations for the observed similarities below.

**Fig. 4.**
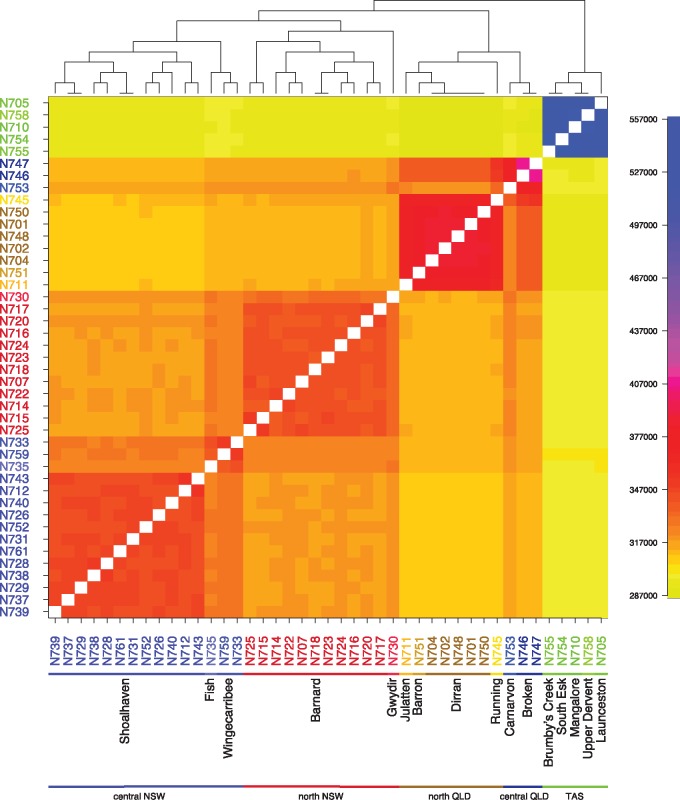
Coancestry matrix from 43 unrelated individuals using FineSTRUCTURE. Each row represents one of the sampled individuals, with the colors along the row for a particular individual representing the number of pieces of their genome for which each other individual shares most recent common ancestry with them. The tree shows the clusters inferred by FineSTRUCTURE from the coancestry matrix. The groupings on the *x*-axis are as in figure 3.

We used FineSTRUCTURE (Lawson et al. 2012) to further investigate population structure and demographic history. This method uses haplotype structure inferred from densely typed markers to infer clusters of individuals with similar patterns of ancestry, and has been shown to be a particularly powerful approach to detecting fine-scale population structure (Leslie et al. 2015). A description of the FineSTRUCTURE algorithm can be found in the Materials and Methods section. Briefly, for each individual, the method first finds the other individuals who share ancestry most closely with it across different regions of the genome. Then, for each individual, the counts of the number of regions sharing most recent ancestry with each other individual form the rows of what is called a coancestry matrix. This information is then used to produce clusters of individuals with similar patterns of ancestry. Figure 4 shows the coancestry matrix and tree inferred by running FineSTRUCTURE on the 43 unrelated samples. The block structure of the coancestry matrix shows that there is strong population structure that is consistent with the samples’ geographic locations, but also shows evidence of finer-scale population structure within each river system.

The deepest branch on the tree separates the Tasmanian samples from the mainland, and the coancestry matrix shows little evidence of the sharing of most recent common ancestors between Tasmania and the mainland, implying a largely distinct population history for the Tasmanian samples, at least over the timescales during which they share recent ancestry with each other. The next branching splits the mainland samples into Queensland and New South Wales clusters, with a further split separating central NSW (including the Shoalhaven, Wingecarribee, and Fish River samples) and north NSW (including the Gwydir River and Barnard samples). However, the Wingecarribee and Fish River samples show more haplotype sharing with the north NSW samples than with the Shoalhaven samples, supporting the evidence from STRUCTURE and PCA that these samples fall between the two larger clusters.

Fine-scale population structure is also evident within some river systems, with the samples from Shoalhaven and Barnard rivers subdivided into smaller population clusters. By contrast, the five samples from the Dirran River in north QLD form a single cluster. The single sample from the Carnarvon River (N753), while showing the greatest level with the other central QLD sample from the Broken River, also shows more sharing with the samples from NSW and less with the samples from north QLD than would be expected based on geography, as they are closer to the north QLD rivers than those in NSW. By contrast, the Broken River samples show much greater sharing with the north QLD samples than the Barnard River samples, as expected. We hypothesize that this may be due to ancestral admixture between the Broken and Carnarvon rivers, and subsequent admixture between the north QLD samples and the Broken River samples only.

We further investigated demographic history using the pairwise sequentially Markovian coalescent (PSMC) method of Li and Durbin (2011). PSMC examines how the local density of heterozygous sites changes along the genome, reflecting chromosomal segments of constant time to the most recent common ancestor (*T*_MRCA_), separated by recombination events. Knowing the coalescence rate in a particular epoch allows estimation of *N*_e_ at that time. As has become clear from applications in other contexts (Prado-Martinez et al. 2013; Wallberg et al. 2014; Nadachowska-Brzyska et al. 2016), this comparison of the two chromosomes within a diploid genome offers an extremely powerful tool for inferring historical effective population size. The power of this approach lies in the fact that there are many thousands of “replicate” segments within a single diploid genome, and these collectively provide precise estimates of historical population size, except for the very recent past and the distant past.

**Fig. 5.**
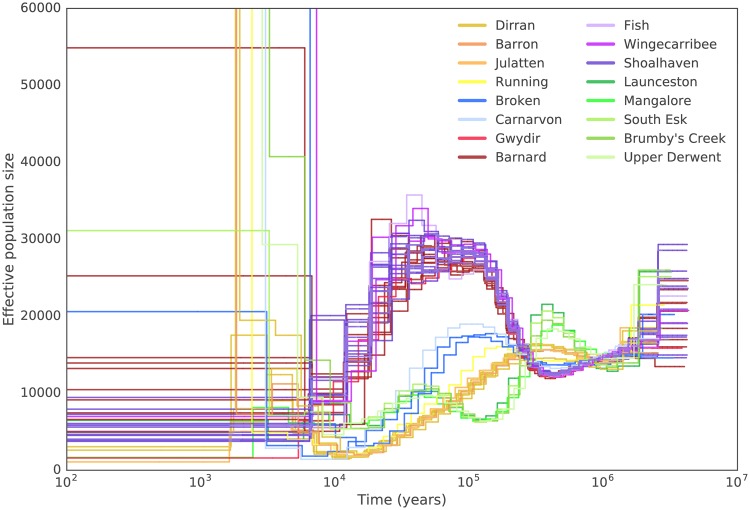
Historical effective population sizes inferred using PSMC. Each line represents a single individual with lines colored according to sampling location. Trajectories were scaled using *g* = 10 and *μ* = 7 × 10^−9^. Effective population size was truncated at 60,000. Samples from a similar sampling location show very similar trajectories.

Figure 5 shows estimates of the effective population size, *N*_e_, from each sample at a series of time intervals. The scaling on the *X*-axis of this plot depends on the generation time, *g*, and the scaling of both the *X*- and *Y*-axes depend on the mutation rate *μ*. Little is known about these two parameters for the platypus, but changing them will affect the estimates for all samples equally, by simply linearly rescaling the axes (supplementary figs. S7 and S8, Supplementary Material online). We will thus focus primarily on conclusions based on relative differences between PSMC estimates as these do not depend on assumptions about *g* and *μ*. For scaling the axes in figure 5, we used *g* = 10 years, following Furlan et al. (2012), which is consistent with the known observations that platypus can live up to 20 years in the wild and that both sexes can reproduce from the age of 2 years, although first breeding in some females can be later than this age (Grant et al. 2004; Grant 2007). In figure 5, we used a mutation rate of 7 × 10^−^^9^/bp/year, the de novo mutation rate we estimated from our own data using the quartet. Note that, in the time-scaling used in figure 5, the method is not informative more recently than ∼10,000 years ago, or further into the past than ∼1–2 My.

Individuals from the same river show strikingly similar trajectories in figure 5, supporting the precision of the relative *N*_e_ estimates and giving us confidence that we are measuring real features of population history. Bootstrapping performed according to the method in Li and Durbin (2011) shows similar trajectories over 100 replicates for each sample (supplementary fig. S9, Supplementary Material online) with the exception of very recent and very distant time points (more recent than 5–10,000 years and older than 1 My), as expected (Li and Durbin 2011). The highly congruent trajectories within a river system suggest that, looking backward in time, the ancestors of these samples were probably part of the same population within the timeframe accessible to the method. Although samples from the same population would be expected to show the same *N*_e_ trajectory, having the same *N*_e_ trajectory does not necessarily mean that samples are from the same population. However, having different *N*_e_ trajectories around a certain time in the past is difficult to reconcile with the individuals’ ancestors coming from the same ancestral population at that time.

One striking feature of figure 5 is that there are clearly four distinct groups of samples (all NSW, central QLD, north QLD, and Tasmania, respectively) with the ancestors of each group clearly having separate population histories until well into the past. It is ∼1 My (in the time-scaling of fig. 5) before NSW, north QLD, and Tasmania begin to share ancestral history, and perhaps 300,000 years until the central QLD and NSW samples might share ancestral history. This implies that there has been extensive population structure in platypus samples across Australia over a long time period.

A second feature of figure 5 is that all *N*_e_ trajectories take their lowest values at the most recent time points for which the method is informative. This is consistent with a decline in platypus numbers across Australia over the time period accessible to the method. In the case of the north QLD samples, the *N*_e_ level becomes extremely low and remains so, and corresponds to a marked population bottleneck (∼10,000 years ago in this time-scaling). This is consistent with the low nucleotide diversity observed in these populations, and with these samples clustering as a single homogenous population in the FineSTRUCTURE results. The central QLD population may well also have been affected by a recent bottleneck and shows very low *N*_e_ during this period.

## Discussion

We have described the first population-scale whole-genome sequencing study of the platypus. The analyses presented here provide insights into the population structure and levels of diversity in this species not previously possible with microsatellite markers or mtDNA. Table 1 provides details of the source and extent of genetic variation used in this and previous studies of platypus demography and population structure.

Our whole-genome data allowed us to estimate relatedness between individuals, and we found that more than half of our samples had a least a third-degree relative among the other individuals sampled from the same river. The quartet samples were all collected within a small distance of each other over a relatively short timeframe (< 3 years). These observations are consistent with an underlying pattern of limited dispersal of (at least some) relatives. This is somewhat surprising given that previous studies using mark-recapture approaches in the Shoalhaven River (Grant 2004; Bino et al. 2015) and other streams (Serena and Williams 2012; Serena et al. 2014) have reported the dispersal of a high proportion of juveniles, especially males, few recaptures of adult males, and the continued capture of unmarked males and females. Of all the pairs of relatives we sampled at the same site, most were female–female or male–female, and not male–male (there are only three male–male relative pairs in supplementary table S4, Supplementary Material online), which is consistent with male-biased dispersal.

Since it appears to be common to collect related individuals when sampling at the same location across several years, it is likely that previous population genetic studies on the platypus (Kolomyjec et al. 2009; Gongora et al. 2012; Furlan et al. 2013) included relatives, but that this was not detected due to the small number of markers used. Some of these studies included individuals in our sequencing study. It is unclear whether and to what extent the results from these earlier studies, particularly those from model-based approaches, would have been affected by the inadvertent inclusion of close relatives.

The inclusion of a quartet in our samples allowed us to estimate the de novo mutation rate in the platypus, the first estimate in a nonplacental mammal. Although our estimates were limited to only a single quartet sequenced to moderate coverage, our estimate is consistent with previous work to estimate mutation rates in mammals. Our point estimate of the rate of 7.0 × 10^−^^9^ (95% CI 4.1 × 10^−^^9^–1.2 × 10^−^^8^/bp/generation) is lower than the estimated rate of 1.2 × 10^−^^8^ in humans and chimpanzees (Kong et al. 2012; Venn et al. 2014) but higher than the rates estimated for laboratory mice (5.4 × 10^−^^9^) (Uchimura et al. 2015). The relative ordering of the point estimates is consistent with the observation that mutation rates in mammals are negatively correlated with body mass and generation time (Welch et al. 2008), but given the various sources of uncertainty in our estimate (some of which are not easy to quantify) it would not be appropriate to place undue weight on it.

For population genetic analyses, descriptive approaches have some advantages over those based on population genetic models. Where descriptive approaches point to clear conclusions, there can be confidence that these are valid. Methods based on population genetics modeling can be more powerful, but their interpretation is always complicated by the fact that they depend, often to a degree which is hard to assess, on the underlying model assumptions. We have thus focused primarily on approaches which do not rely on population genetics modeling. (Some of the approaches used above, e.g., STRUCTURE, and FineSTRUCTURE, do involve statistical models which aim to capture features of the data, but none is based on models of historical population dynamics or history.) Our analyses confirm the strong structure previously reported in the species (Gongora et al. 2012; Furlan et al. 2013), with both pronounced differentiation on the mainland in a north–south direction, and separation of the Tasmanian samples from all other groups. Consistent with Furlan et al. (2013), we saw little evidence for structure within the Tasmanian samples, which could be due to greater overland migration in this wetter climate. On the other hand, the apparent lack of structure could simply be because of our small sample size (6 individuals).

Our results suggest several instances of higher genetic similarity between individuals than expected given their sampling locations. For example, the Fish and Wingecarribee samples come from rivers that flow into different systems on either side of the Great Dividing Range (the west-flowing Murray-Darling system and the east-flowing Hawkesbury system), and yet look extremely similar to one another on the PCA and in STRUCTURE, and are grouped together in the FineSTRUCTURE analysis. This finding is consistent with Furlan et al. (2013), who also reported that there was little or no genetic differentiation in mtDNA and microsatellite markers between platypus either side of the Great Dividing Range in Victoria. This genetic similarity might be due to overland migration or historical connections between the Murray-Darling and Hawkesbury systems that have been altered due to climate or geological changes. The similarity between the sample from Rifle Creek, which is part of the west-flowing Mitchell River system, and those from east-flowing Dirran Creek and Barron River, could probably be explained by occasional overland dispersal. Although they are part of different drainages, the sampling locations are all within 100 km, and the climate is much wetter, so migration may have been possible if the ephemeral streams and refuge pools in the area persisted for long enough. The similarity may also be due to the population decline evident from the PSMC results, and low genetic diversity across the north QLD samples as a whole.

It is interesting that Kolomyjec et al. (2009) reported that 13 of the 120 individuals they analyzed at 12 microsatellites appeared to be first-generation migrants from the Shoalhaven to the Hawkesbury River systems (the latter including Wingecarribee), or vice versa. (Where sampling includes close relatives, as seems possible in the light of our results, estimating numbers of migrants may not be straightforward.) We did not find any evidence of migrants nor of recent admixture (using the PCA or STRUCTURE) between the Shoalhaven and the Wingecarribee, consistent with the different river systems being separated by steep terrain along much of their border, despite the physically close sampling locations. It may be that we simply did not happen to sample such individuals, or that the sampling locations were further apart than those of Kolomyjec et al. (2009).

The PSMC results provide a glimpse of the past demographic histories of the different sampling locations, which was not possible in previous studies based on a small number of markers. The fact that these groups show such different histories emphasizes that running this method on a single sample from a species might not provide a representative picture of the coalescence process for that species, as noted by Nadachowska-Brzyska et al. (2016) in a study on flycatchers. Our PSMC results reveal a recent reduction in *N*_e_ in all regions, with a particularly low *N*_e_ in the Queensland samples.

The Queensland bottleneck likely reflects the historical and current isolation and paucity of suitable habitat for platypus between North (Australian Wet Tropics) and Central QLD, known as the “Burdekin gap” (named for the Burdekin River). This hot and dry area is currently climatically unsuitable for platypus (supplementary fig. S15, Supplementary Material online) and has long acted as a barrier to genetic exchange (James and Moritz 2000; Schäuble and Moritz 2001). The declining *N*_e_ but separate trajectories of the north and south QLD samples reflect accumulating evidence of the impact of arid periods—glacial maxima—on the mesic forest biotas of the region (Bryant and Krosch 2016). South of this, the Broken River is within the mideast QLD diversity hotspot for rainforest faunas, and the nearby coast has functioned as a small and isolated climate change refugium since the Last Glacial Maximum. Carnarvon Gorge is an oasis in the semiarid heart of central QLD, making it the only suitable current habitat for platypus for hundreds of kilometres (supplementary fig. S15, Supplementary Material online). The possible ancestral admixture between the Carnarvon and Broken river systems, and subsequently between the Broken River and other north QLD samples, is hard to reconcile with the current climate of these areas. The high level of homozygosity seen in the Carnarvon sample may reflect the low effective population size over the last ∼50,000 years (fig. 5) or may be the result of recent inbreeding. Regardless, it suggests that the Carnarvon River platypuses may be particularly vulnerable and should therefore be a priority for conservation, as noted by Kolomyjec et al. (2013).

In contrast to the QLD samples, the NSW individuals appear to have had higher and relatively stable *N*_e_ over much of their history, with population decline only in more recent time periods. The high *N*_e_ makes sense given recent paleoecological evidence that parts of this region remained wetter (relative to North QLD and Southeast Australia) during the Last Glacial Maximum (Moss et al. 2012) and, based on paleoclimate modeling, the region is inferred to have been a large, stable mesic refuge over the past 120,000 years (Weber et al. 2014; Rosauer et al. 2015). Determining whether the recent decline in *N*_e_ is due to late Pleistocene climate change or other causes would require high resolution modeling of paleoclimates for this region.

PSMC results can be used to infer when different populations separated by noting where the *N*_e_ estimates start to diverge when moving forward in time (right to left in fig. 5) (Li and Durbin 2011; Nadachowska-Brzyska et al. 2013; Thomas et al. 2015). Regardless of the scaling of the axes, figure 5 implies that all of the NSW samples share an ancestral population more recently than any of them shares an ancestral population with any of the other samples. Looking backward in time, the central QLD samples next share an ancestral population with the NSW samples, before this larger group share an ancestral population with either the north QLD or Tasmanian samples. There are slight differences in *N*_e_ trajectories which persist throughout the timescales covered by figure 5. (As noted above, we would expect, and see, differences at the extreme right of the figure reflecting noise associated with the loss of precision of the estimates in the ancient past.) Between ∼1 and 2 Ma in the time-scaling of the figure, the *N*_e_ trajectories of each of the Tasmanian, North QLD, NSW, and Central QLD samples are extremely close, but not identical. Further, the differences are typically larger between than within these three groupings. One explanation for this is that all groupings do share an ancestral population by this time, but that there are systematic differences between the estimates from each which result in the small differences in *N*_e_ trajectories between groups. The other explanation is that the groupings do not share an ancestral population at that time, but instead their ancestral populations just happened to have extremely similar population sizes. This second explanation would be consistent with the divergence of *N*_e_ estimates on the right of figure 5, where the values for the north QLD and Tasmanian samples jump to higher values (looking left to right) before those from NSW/central QLD. In our view, limited weight should be put on this last observation because *N*_e_ estimates are noisy at the right of the picture, and the nature of this noise could differ systematically between groupings for many reasons, including systematic differences in properties of SNPs in those groups. Our data cannot distinguish between these two explanations (sharing of an ancestral population by all samples, or distinct ancestral populations with extremely similar *N*_e_ values over long time periods), but the former explanation is more parsimonious and appears to us to be the more likely.

Regardless of the preferred explanation of the two in the previous paragraph, there is no evidence in our data that the north QLD, central QLD, and NSW samples shared an ancestral population before either shared an ancestral population with the Tasmanian samples. Under the first explanation, all three groupings first shared an ancestral population around the same time. Under the second explanation, none of them share an ancestral population over the time scales for which reliable PSMC *N*_e_ estimates are available. We think it is most likely that there were three ancestral populations (TAS, north QLD, and north NSW/central QLD) which all coalesced around the same time (∼800 KYA, using the scaling in fig. 5). This is hard to reconcile with the phylogenetic tree inferred by Gongora et al. (2012) using mitochondrial data, in which all north and central QLD samples (including from Dirran Creek, Running River, and Carnarvon) coalesce much more recently with each other than they do with any other population. On the other hand, the wide 95% credible intervals for divergence times inferred by Gongora et al. (2012) overlap between the different events, and our estimates from figure 5 fall within them. Although mitochondrial ancestries could indeed differ from population ancestries and those of autosomal loci, the estimates from PSMC are expected to be more precise than those available from any single locus, since the PSMC method, in effect, averages over every locus in the genome.

Interestingly, the divergence times we and Gongora et al. (2012) have estimated predate the earliest fossil evidence for platypus (Musser 1998, 2013), although we are conscious that our absolute estimates do depend on the generation time and mutation rate. This finding does not necessarily contradict fossil evidence but suggest that the modern platypus extends back to the Early to Middle Pliocene. This could be consistent with it having evolved from the giant platypus species *O. tharalkooschild*, as suggested by Pian et al. (2013).

We have presented the first whole-genome resequencing study of the platypus. Our results have provided insights into the strong population structure in this species as well as the demographic history of the different sampling locations, at a level that was impossible with older molecular tools. Our analyses also identify certain populations (notably, Carnarvon) with particularly low diversity, and could be used to set priorities for conservation efforts, especially given the increasing threats to platypus habitat due to climate change. Future studies are likely to shed further light on the population history and biology of this fascinating species. This study emphasizes the power of whole-genome sequencing for inference of population dynamics and historical demography, even where sampling of individuals is constrained.

## Materials and Methods

### Samples and Sequencing

We obtained DNA samples from 61 platypuses, extracted from toe webbing, spleen, liver, or cell lines. These included between 1 and 18 individuals from each of 17 waterways across most of the platypus range, excluding Victoria because no high-quality DNA samples were available. For quality control, we included a duplicate sample from Dirran Creek, north QLD, and a father–daughter pair obtained from Taronga Zoo (the father and mother originally being from the Fish River in the Macquarie Basin in NSW). These were sequenced in four tranches. Details of the library preparation and paired-end (PE) sequencing are shown in Table S6. Three samples were discarded because they failed sequencing quality control. Mean coverage and insert size for the remaining 58 samples are shown in supplementary figure S16 and table S2, Supplementary Material online.

### Reference Genome Reassembly

We used an improved genome assembly produced in collaboration with the G10K consortium, based on Pacific Biosciences long read sequencing and Dovetail Hi-C scaffolding (Bioproject reference PRJNA433451; Genbank accession GCA_002966995.1), which we will call ornAna3 here.

### Mapping and Variant Calling

We mapped the paired-end data from all 58 samples to ornAna3 using Stampy (Lunter and Goodson 2011) without BWA premapping. Duplicates were removed using Picard MarkDuplicates tool (http://broadinstitute.github.io/picard). Variant calling was performed jointly on the 58 samples using the PLATYPUS variant caller (Rimmer et al. 2014), and we obtained a total of 14,127,611 biallelic SNPs with the “PASS” filter. We removed indel calls, monomorphic positions, and 2042 positions where the reference individual (N720) was called homozygous for the alternative allele, which may represent errors in the reference genome. Inspection of the variant calls which were discordant between duplicate samples (see supplementary section S2, Supplementary Material online) revealed that PLATYPUS incorrectly called positions as heterozygous despite no reads supporting the variant at that position. We thus removed 223,103 variants across all individuals studied for which PLATYPUS reported no reads supporting the variant. We assessed sites for Hardy–Weinberg Equilibrium, as implemented in VCFtools (0.1.14), and excluded SNPs with *P* value <10^−^^7^. These variants may be errors but the substantial population structure in our samples may also cause variants to fail this test. We filtered out variants with quality <60, and kept only SNPs with no missing data. This SNP set forms the stringent SNP set referenced in the results.

To assign contigs to chromosomes, we took advantage of the chromosome assignment made for the ornAna1 reference, in which sequences where attributed to chromosomes 1 to 7, 10–12, 14, 15, 17, 18, 20, X1, X2, X3, and X5. Specifically, we broke down each chromosome from ornAna1 in pieces of 500Kb and aligned these pieces to the new reference genome using bwa mem (BWA version 0.7.12). We excluded the pieces that resulted in primary and secondary alignment, because these are likely to be due to mis-assemblies in ornAna1. For the remaining pieces, we kept only the primary alignment with mapping quality of 60 (highest value). A total of 304 contigs, with total sequence length of 1,779,183,769 bp, had at least one piece of ornAna1 chromosome aligning to them with these criteria. The remaining 4268 contigs (211,276,236 bp) were excluded from further chromosome assignment using homology to ornAna1. For each contig, we computed the number of base pairs covered by pieces coming from each of the ornAna1 chromosomes. We assigned the ornAna1 chromosome label to a contig when more than 90% of covered sequence within the contig was attributed to the chromosome in ornAna1 and when more than 10% of the total sequence of the contig was covered by ornAna1 sequence pieces. Finally, we compared the coverage for each contig between males and females to validate the autosomal contigs. For subsequent analyses, we retained only 54 assigned autosomal contigs with at least 50 SNPs that passed our filters, covering 965,354,475 bp in total. This final SNP callset includes a total of 6,727,617 SNPs.

We determined the callable regions of the genome by running PLATYPUS with the callRefBlocks option to determine the bases where a good quality reference call could be made, and combined these with the SNP callset positions to give a callable genome length of 910,563,690 bp.

### Identifying Relatives

To identify relatives among the sampled individuals, we ran the KING algorithm (Manichaikul et al. 2010) as implemented in VCFtools (0.1.14) (Danecek et al. 2011), which uses the number of SNPs that are identical-by-state 0, 1, or 2 to estimate the kinship coefficient. For each broad sampling location (north QLD, north NSW, central NSW, and Tasmania) we removed SNPs with minor allele frequency (MAF) < 0.05 across samples from that location. For several of the population genetic analyses described below (*F*_ST_ and nucleotide diversity calculations; STRUCTURE; FineSTRUCTURE), we removed one individual from each relative pair (up to and including third-degree relatives), leaving 43 individuals (shown in blue in Table S1). Specifically, we removed N713, N719, and N721 from the Barnard River; N703 and its duplicate, N749, from Dirran Creek; N727, N734, N741, N742, N757, and N760 from the Shoalhaven River; N732 and N744 from the Wingecarribee River; N736, the daughter from the Fish River (Taronga Zoo); and N756 from Brumby’s Lake in Tasmania.

### Long Runs of Homozygosity

We followed the approach of Prado-Martinez et al. (2013) to detect long regions of homozygosity (LROHs). For each sample, we calculated the heterozygosity (i.e., proportion of callable sites that were called as heterozygous) in overlapping windows along the genome, using the set of callable sites defined above. We examined the distribution across windows, selected a threshold (we chose 5 × 10^−^^5^, based on the local minima in supplementary fig. S17, Supplementary Material online) below which a window was classed as “homozygous,” then merged overlapping homozygous windows. We used 1 Mb windows, shifted by 200 kb each time, and used only the scaffolds classed as autosomal that were longer than 1 Mb (total length 963.8 Mb). We then calculated the proportion of the examined genome that was in homozygous chunks, *F*_ROH_, as a measure of inbreeding (supplementary fig. S2, Supplementary Material online).

### Population Genetic Analyses

#### Nucleotide Diversity, F_ST_ Estimates, PCA and STRUCTURE

We used VCFtools (0.1.14) to compute *π* per site --site-pi on all SNPs in unrelated individuals from each sampling location. The total nucleotide diversity for a sampling location was computed by summing over values of *π* for all polymorphic SNPs and dividing by the total number of callable sites (910,563,690).

We used EIGENSOFT (v6.0.1) (Patterson et al. 2006) to estimate *F*_ST_ between sampling locations and to run a Principal Components Analysis (PCA). We ran STRUCTURE (Pritchard et al. 2000) with the admixture model for *k* = 2, 3,…10, with a burn-in of 20000 followed by 50000 MCMC repetitions, repeating each for five replications.

For these analyses, we took the callset on the unrelated samples and removed SNPs with MAF < 0.05 (leaving 3,245,503 SNPs), then carried out LD pruning using PLINK (Purcell et al. 2007). Specifically, for windows of 50 SNPs, we removed one of each pair of SNPs if the *r*^2^ between them was greater than 0.1, and then shifted the window five SNPs forward (PLINK option: --indep-pairwise 50 5 0.1). This left us with 128,803 SNPs.

#### Phasing and FineSTRUCTURE

We used SHAPEIT2 (O’Connell et al. 2014) to phase haplotypes across samples. SHAPEIT2 was run on the full cleaned SNP set (i.e., before MAF filtering and LD pruning) using a window size of 0.5 Mb, 200 conditioning states, and 30 iterations of the main MCMC, and incorporating phase-informative sequencing reads to improve phasing at rare variants (Delaneau et al. 2013). The haplotypes were postprocessed with duoHMM (O’Connell et al. 2014), using the two known pedigrees in the sample set to improve the phasing and correct Mendelian errors.

The phased haplotypes were used to run FineSTRUCTURE (Lawson et al. 2012). The FineSTRUCTURE algorithm involves two separate stages. The first stage considers each sampled individual separately, and proceeds along the phased chromosomes (or, in our case, scaffolds) in each individual. For a particular individual, the method partitions the chromosome into pieces, and for each such piece it searches among the other sampled individuals to find the one who shares most recent common ancestry for that part of the chromosome. (The partitioning of the phased chromosomes into pieces, and the identification of the piece in another individual sharing most recent common ancestry, are undertaken jointly in the algorithm.) For this individual, one can then count the number of chromosomal pieces for which each other individual shares most recent common ancestry. This process is undertaken separately for each sampled individual. These shared ancestry counts can be visualized in what is called a coancestry matrix. The second stage of the FineSTRUCTURE algorithm involves taking these coancestry counts for each individual, and forming clusters of individuals with the property that individuals within the same cluster have similar patterns of sharing with other individuals. One can then obtain a tree relating the sampled individuals by successively merging clusters which are similar. (When FineSTRUCTURE produces the tree, similarity of clusters is defined in terms of changes in the likelihood of the statistical model used by the algorithm to infer clusters. The tree should not be interpreted as a direct estimate of the ancestral history of the samples.)

FineSTRUCTURE was run using the linked model with a uniform recombination rate and default parameters except 2 M iterations of the MCMC (1 M for burn-in), 200,000 tree iterations, and starting *N*_e_ of 10 M to avoid the EM algorithm finding a local optima with zero recombination. Three MCMC runs were performed, giving identical sample clustering.

#### Pairwise Sequentially Markovian Coalescent

We ran PSMC to investigate historical population sizes (Li and Durbin 2011). A diploid fasta file was created from the SNP set referenced above, and used to run PSMC for each sample. PSMC was run for 25 iterations using an initial *θ*∕*ρ* ratio of 5 and the default time patterning. Bootstrapping was performed as in Li and Durbin (2011) by resampling 5 Mb chunks of the genome with replacement to generate 30 artifical chromosomes of 100 Mb each and running 100 bootstrap replicates.

#### Habitat Modeling

We used the maximum-entropy approach of Phillips et al. (2006) to model the platypus habitat (supplementary fig. S15, Supplementary Material online). This method uses a set of layers, or environmental variables, as well as a set of geo-referenced occurrence locations, to produce a model of the range of a given species. We used the following variables: Precipitation—annual mean; Temperature—annual max mean; Temperature—annual min mean; Drainage—variability; Drainage—average; Drainage Divisions Level 1; Drainage Divisions Level 2; River Regions.

## Data Availability

Sequence data were deposited at the ENA under study ERP106780. The platypus reference genome is available as BioProject PRJNA433451.

## Supplementary Material

Supplementary data are available at *Molecular Biology and Evolution* online.

## Author Contributions

P.D. and J.G. conceived the study. P.W., T.D., S.K., M.S., T.G., F.G., and J.G. contributed samples and performed experiments. P.P. and R.B. performed library preparation and led the sequencing. H.C.M., E.M.B., and J.H. performed the analyses. H.C.M., E.M.B., J.H., P.D., C.M., and J.G. drafted the paper. P.D. supervised the project. All authors read and commented on the manuscript.

## Supplementary Material

Supplementary Data
