# Supplementary material for: Insights into Platypus Population Structure and History from Whole-Genome Sequencing
Source: Mol Biol Evol. 2018 Mar 20;35(5):1238–52. doi: 10.1093/molbev/msy041 (PMC5913675; doi:10.1093/molbev/msy041)
Supplement: Supplementary Data [file msy041_supp.pdf]

Supplementary Material

Contents

S1 Assessing the new reference genome **2**

S2 Quality control of variant calls **2**

S3 *De novo* mutation rate estimation **2**

S4 Supplementary Figures **4**

S5 Supplementary Tables **22**

S1 Assessing the new reference genome

The new genome assembly, named ornAna3, has 4572 contigs with 476 gaps, a scaffold N50 of 18.72 Mb and covers a total of 1,990.4 Mb of sequence. The new platypus reference genome will be made available no later than the time of publication of this paper. To assess the scaffolding error rate, we examined inheritance patterns in a family quartet (two parents + two offspring) which we found amongst the samples from the Shoalhaven River. Changes in the pattern of inheritance in the two offspring ("switches") represent either crossovers or switch errors. The density of these depends on the number of informative SNPs (i.e. SNPs at which one parent is heterozygous and the other homozygous). For counting the switches, we called variants with PLATYPUS (Rimmer et al., 2014) jointly in the quartet on both ornAna1 and ornAna3, keeping the parameters the same to enable fair comparison between the assemblies. We then identified places where the offspring changed from carrying the same haplotype from a particular parent to carrying a different haplotype, or vice versa. We removed switches that involved fewer than 5 SNPs because these were likely due to genotyping errors. We restricted the analysis to autosomal scaffolds over 50kb for ornAna1 and over 1Mb for ornAna3.

S2 Quality control of variant calls

Analysis with KING confirmed that we had sequenced a duplicate sample (N703 = N749), as well as a whole family quartet (Table S4). We used these to assess the quality of our POPGEN callset. The rate of discordant genotypes between N703 and its duplicate sample N749 was 5.03×10^{-3} per SNP, or 3.75×10^{-5} per callable base (Table S3). The Mendelian error rate in the quartet was 1.1×10^{-3} per SNP, or 8.3×10^{-6} per callable base.

There were 2,042 SNPs at which the reference individual (N720) was called homozygous for the alternative allele. Of these, 344 were also called homozygous for the alternative allele in all samples, suggesting that the reference sequence was incorrect at this position.

S3 *De novo* mutation rate estimation

A subset of the Mendelian errors are *de novo* mutations, and for many population genetic analyses it is useful to know the mutation rate. PLATYPUS (Rimmer et al., 2014) includes a *de novo* variant caller, and we used this caller on the filtered SNP set to find the number of *de novo* mutations for the two offspring in the quartet. To calculate the mutation rate from the number of *de novo* mutations, we determined the callable proportion of the genome using the Platypus reference call blocks as above with an additional filter to restrict to regions covered by ≥ 10 reads, as the PLATYPUS *de novo* variant caller requires 5 supporting reads to call a variant. The candidate *de novo* mutations produced by Platypus were filtered to remove variants already seen in the population, as real *de novo* mutations are unlikely to be circulating in the population.

$$\mu \approx \frac{\text{N. putative } \textit{de novos} \text{ for N742} + \text{N. putative } \textit{de novos} \text{ for N757}}{\text{N. sites callable in N742 \& parents} + \text{N. sites callable in N757 \& parents}}$$
$$\approx \frac{6+6}{880839653 + 830770728} \approx 7.01\times10^{-9}/\text{bp/generation.}$$

We obtained a 95% confidence interval for the *de novo* mutation rate as the set of possible values for the rate λ with the property that we would not reject the null hypothesis that the true rate took the value λ in a 2-side hypothesis test with p=0.05 under a model in which the observed number of *de novo* mutations has a Poisson distribution with mean $= \lambda \times$ the number of sites callable in the parents.

The true mutation rate may be somewhat higher than this, as the sequence coverage for these samples is too low to comprehensively call all heterozygous variants. Unfortunately there was insufficient DNA remaining from the samples in the quartet to try to validate *de novo* mutations.

S4 Supplementary Figures

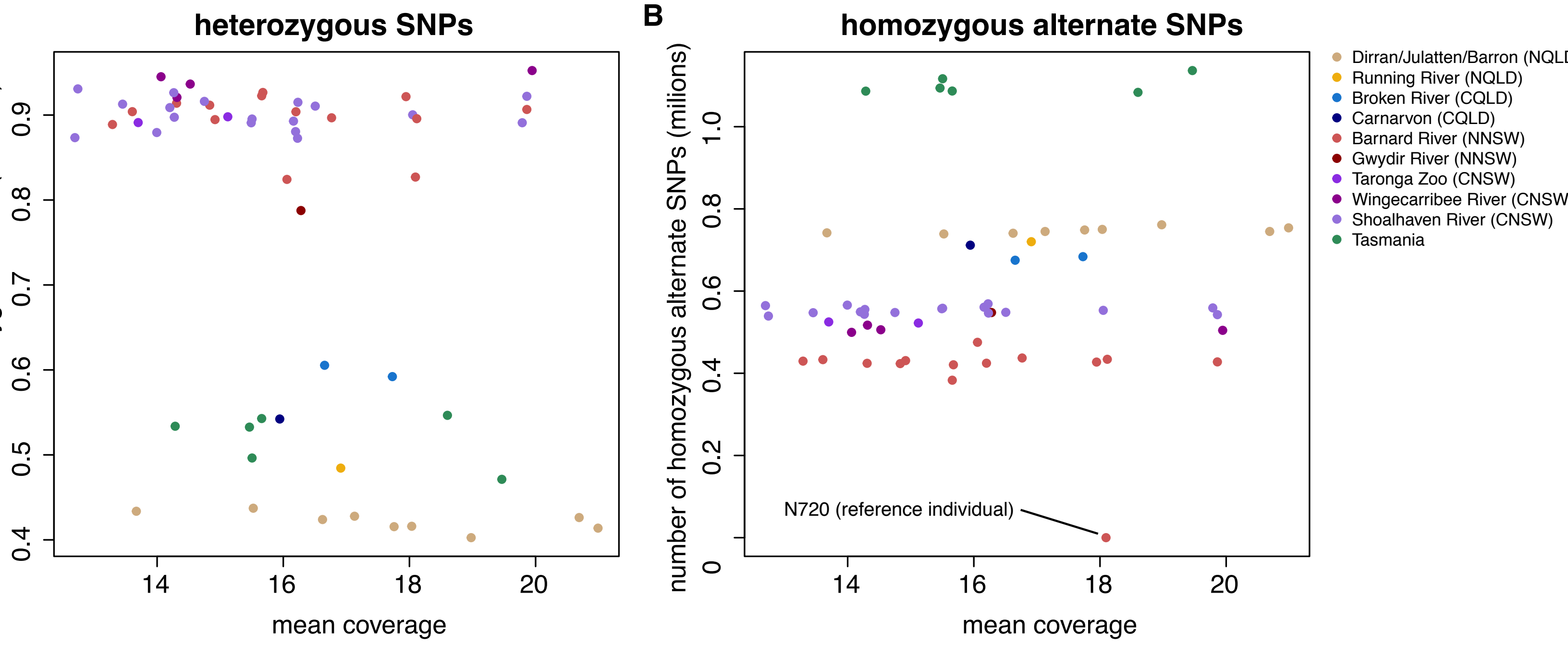

Figure S1: **Number of heterozygous or homozygous variants versus coverage.** Number of heterozygous (A) or homozygous alternate genotypes (B) versus coverage per sample.

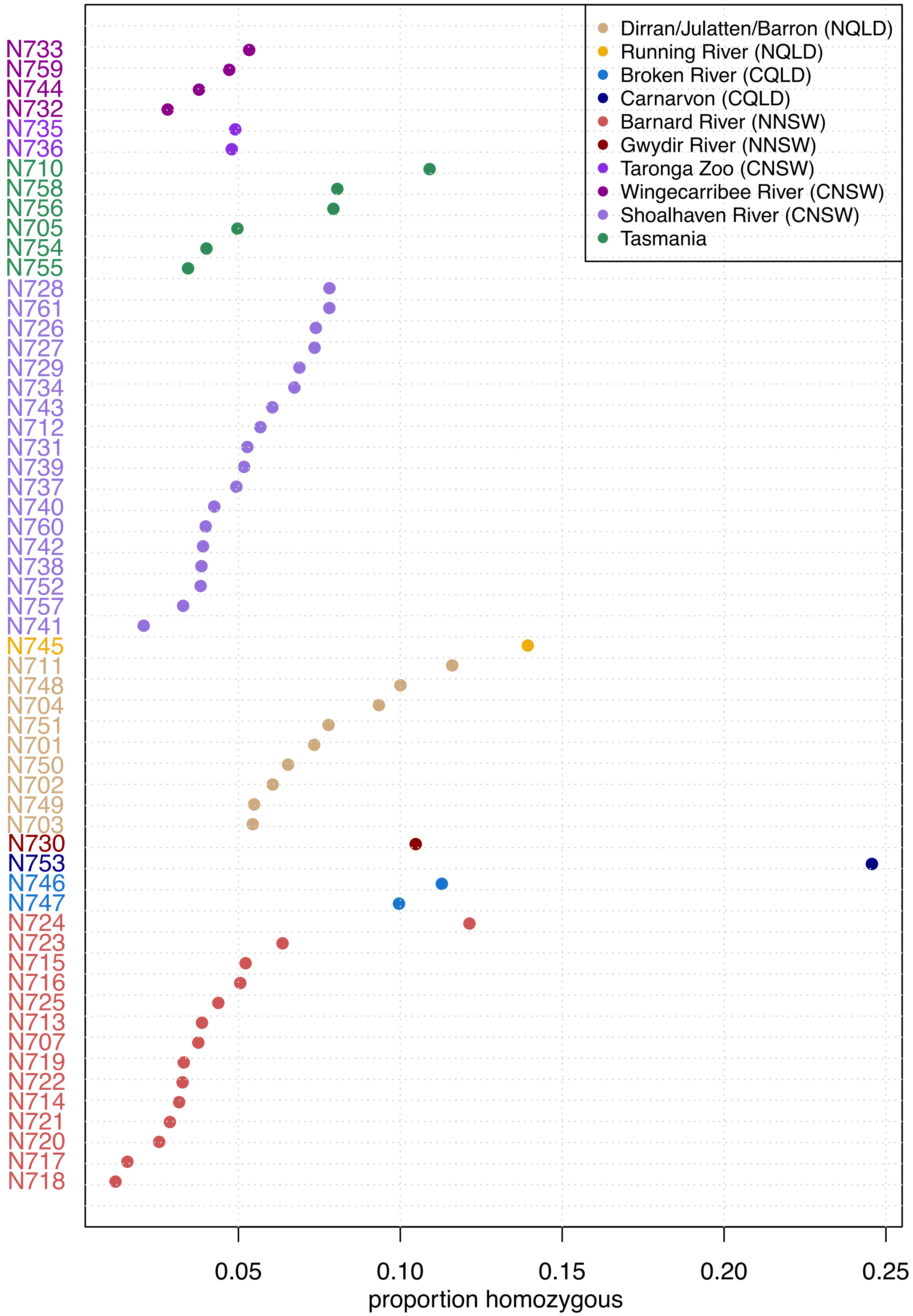

Figure S2: **Summary of overall homozygosity.** The plot shows the proportion of the analysed genome, F_{ROH}, classed as being in homozygous chunks, as described in Methods. We analysed 963.8Mb of autosomal sequence in scaffolds longer then 1Mb. Samples are ordered by sampling location and then by F_{ROH}.

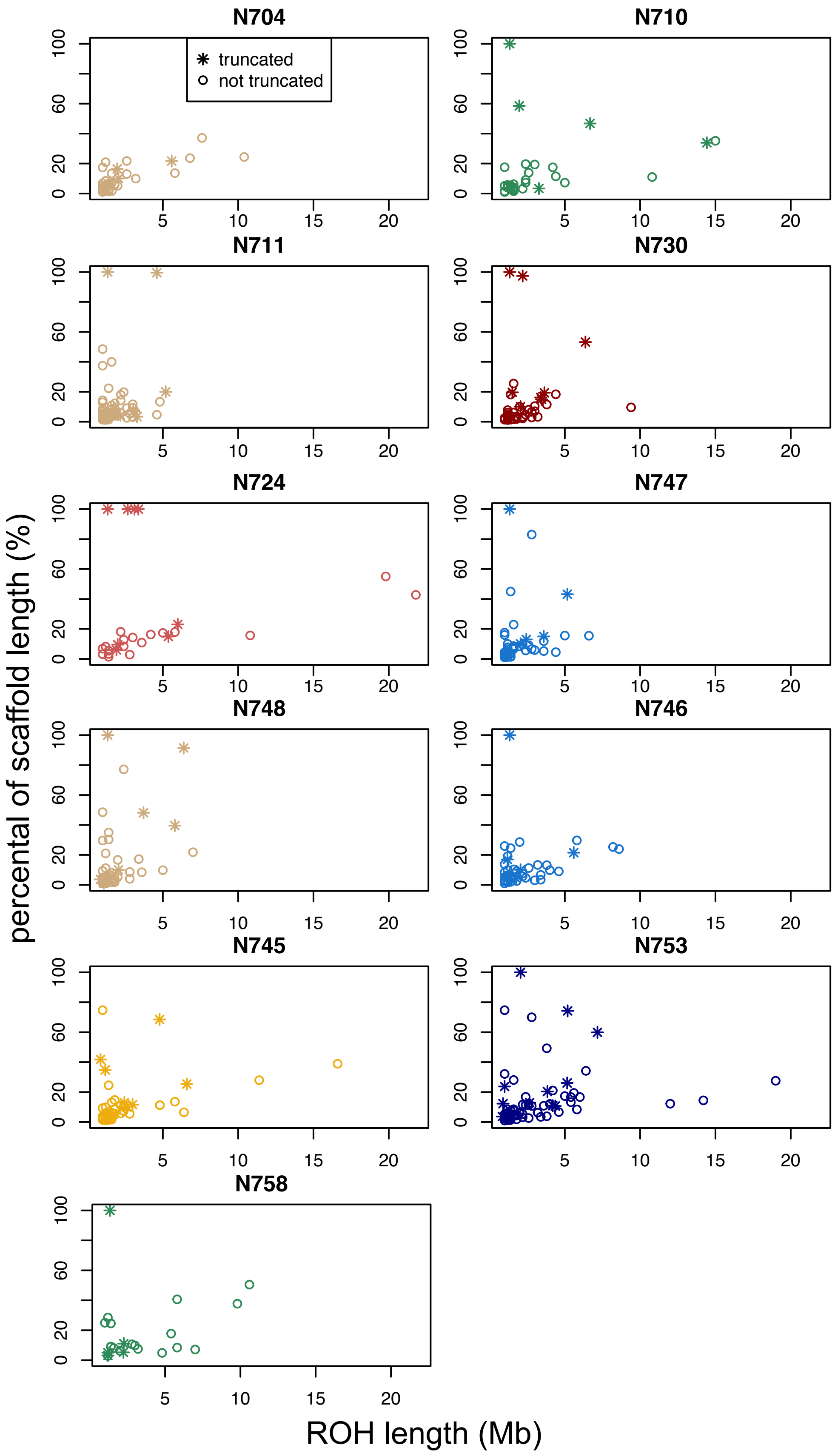

Figure S3: **Some ROHs are truncated by scaffold end or windows with low callability.** The percentage of the scaffold covered by the ROH is plotted against the ROH length, for samples with F_{ROH} over 0.08. The point type indicates whether or not the ROH was truncated either by a scaffold end or by hitting a window which was excluded due to having less than 80% of bases callable (asterisks) or was not artificially truncated (empty circles).

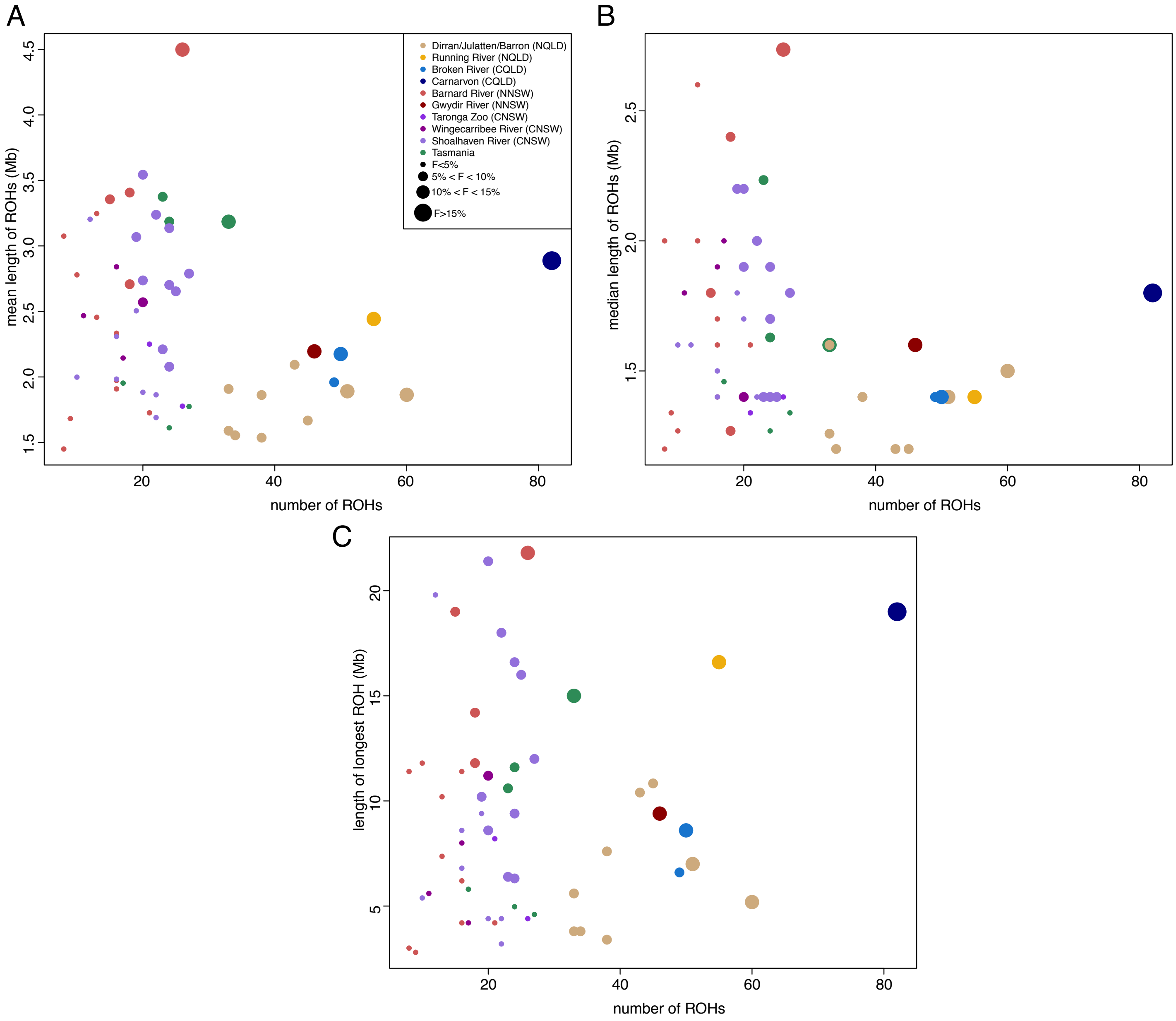

Figure S4: **Summary of length and number of ROHs.** The plots show various summaries of the ROH length per sample plotted against the number of ROHs. A) Mean length; B) Median length; C) Longest ROH.

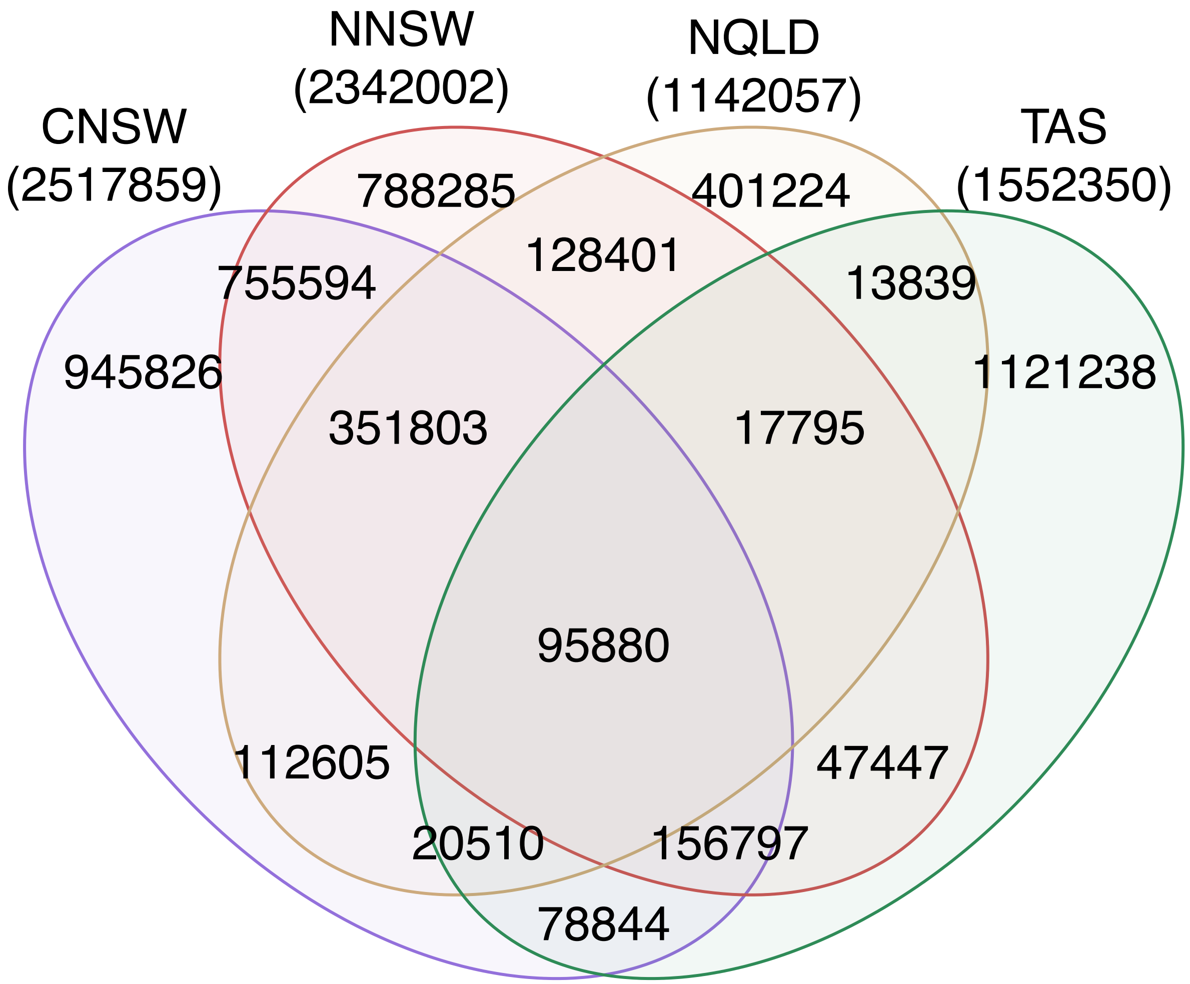

Figure S5: **Venn diagram showing number of SNPs segregating in different sample groupings.** The numbers in parentheses are the total numbers of segregating SNPs in the unrelated samples from that grouping, out of the ~6.7 million SNPs segregating in the 57 samples. Note that five samples have been chosen randomly from the unrelated CNSW, NNSW and NQLD samples, so that the sample size is the same as in Tasmania.

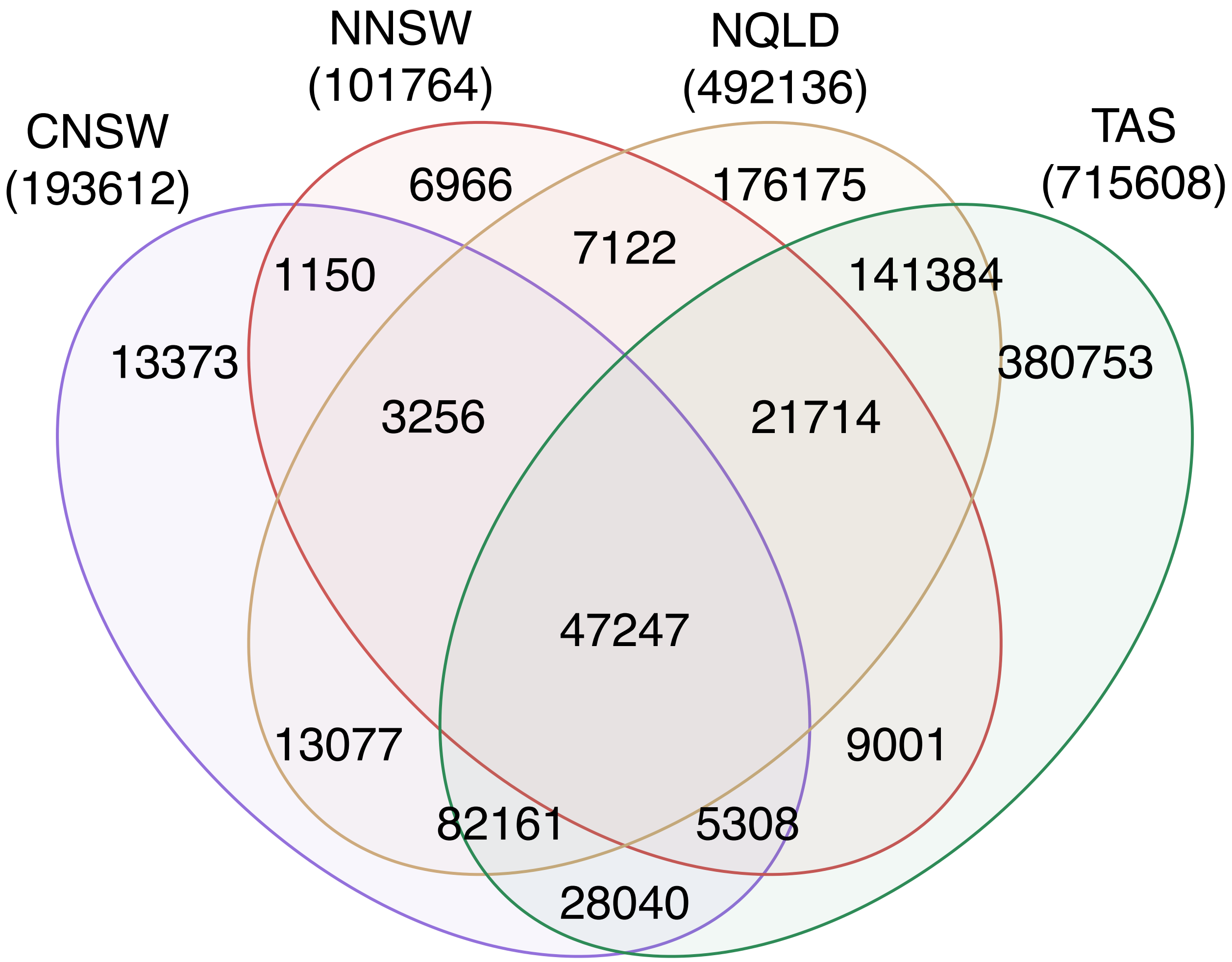

Figure S6: **Venn diagram showing number of fixed differences from the reference in different sample groupings.** The numbers in parentheses are the totals for that grouping, out of the ∼6.7 million SNPs segregating in the 57 samples.

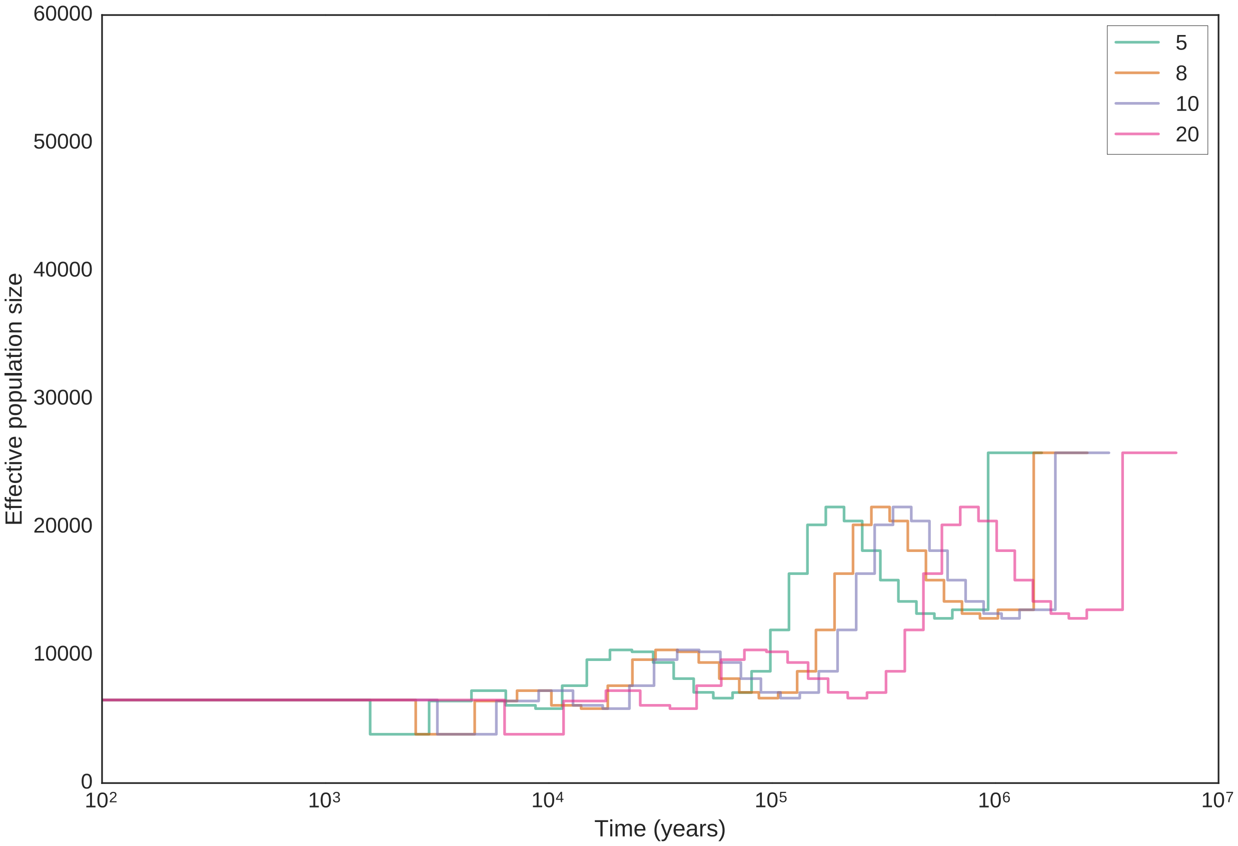

Figure S7: **Effect of generation time on PSMC results.** This plot illustrates how the choice of g used to scale the PSMC output shifts the estimates of N_e along the X axis, using the results from a single sample, N705. Furlan et al. (2012) used $g = 10$, and we also show $g = 5$, $g = 8$ and $g = 20$ here for comparison. Note that varying the generation time affects only the estimated time and has no effect on the effective population size.

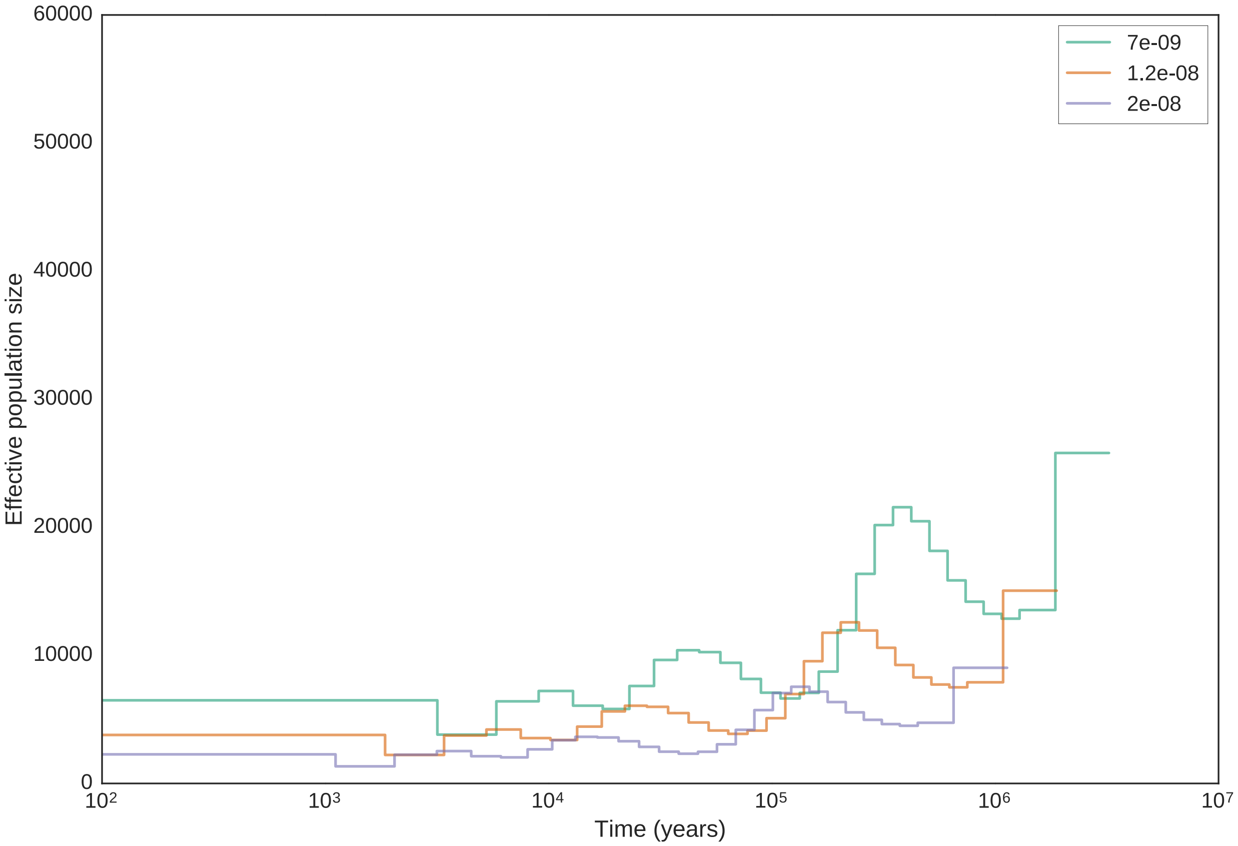

Figure S8: **Effect of mutation rate on PSMC results.** This plot illustrates how the choice of μ used to scale the PSMC output shifts the estimates of N_e along the X and Y axes. We show the results for $\mu = 1.2{\times}10^{-8}$, $\mu = 2{\times}10^{-8}$, and $\mu = 5{\times}10^{-9}$.

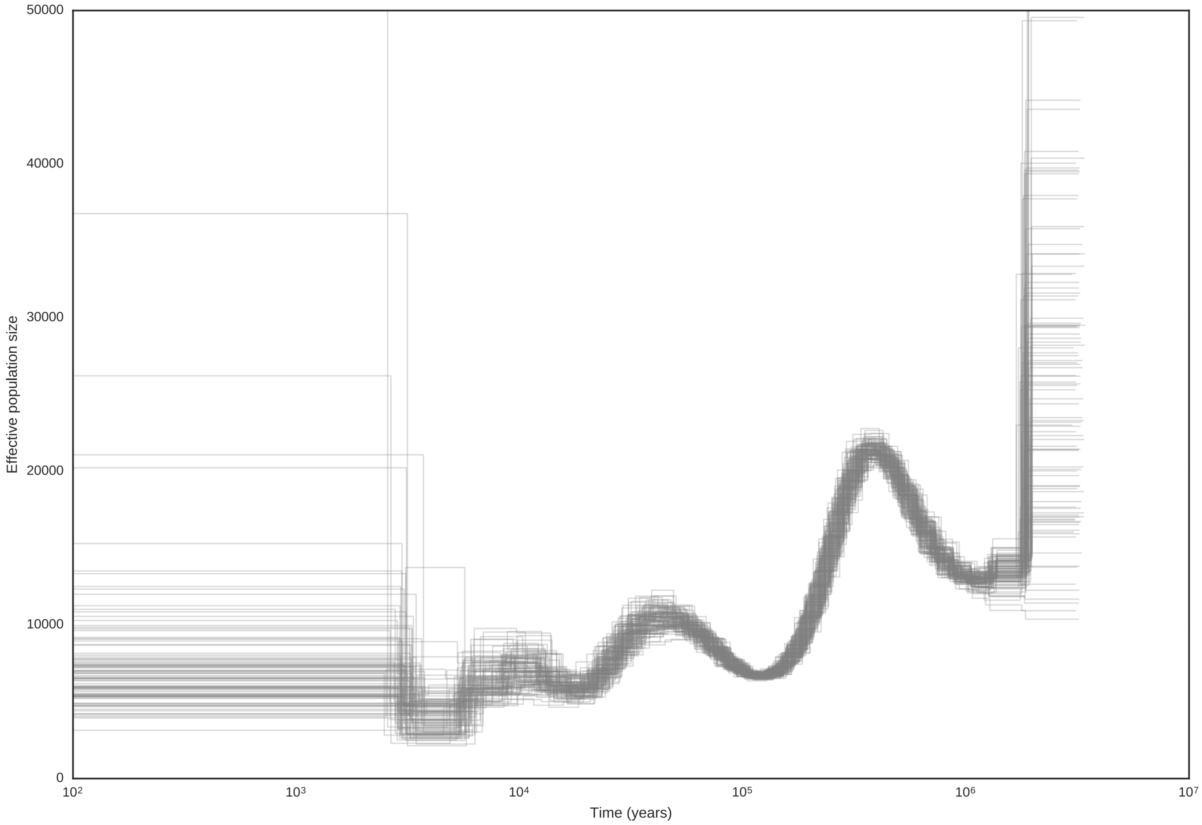

Figure S9: **Bootstrapping of PSMC results.** 5Mb segments of the genome were resampled with replacement 100 times (as in Li and Durbin (2011). The results for sample N705 are shown here. Trajectories were consistent across bootstrap replicates with the exception of time points in the recent past (before 10,000 years) and distant past (before 2,000,000 years). These were scaled using $g = 10$ years and $\mu = 7.0{\times}10^{-9}$.

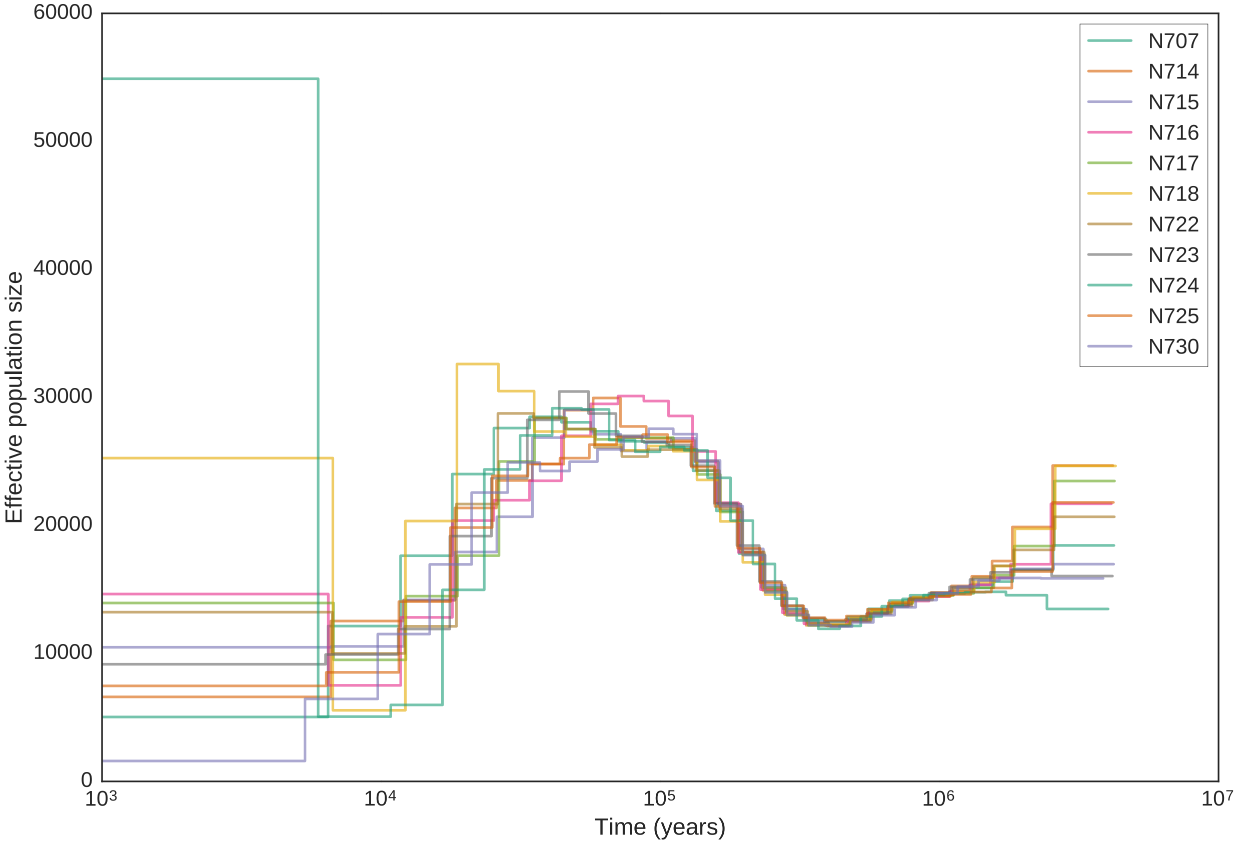

Figure S10: **Historical effective population sizes inferred from north NSW samples using PSMC.** These were scaled using $g = 10$ and $\mu = 7.0{\times}10^{-9}$.

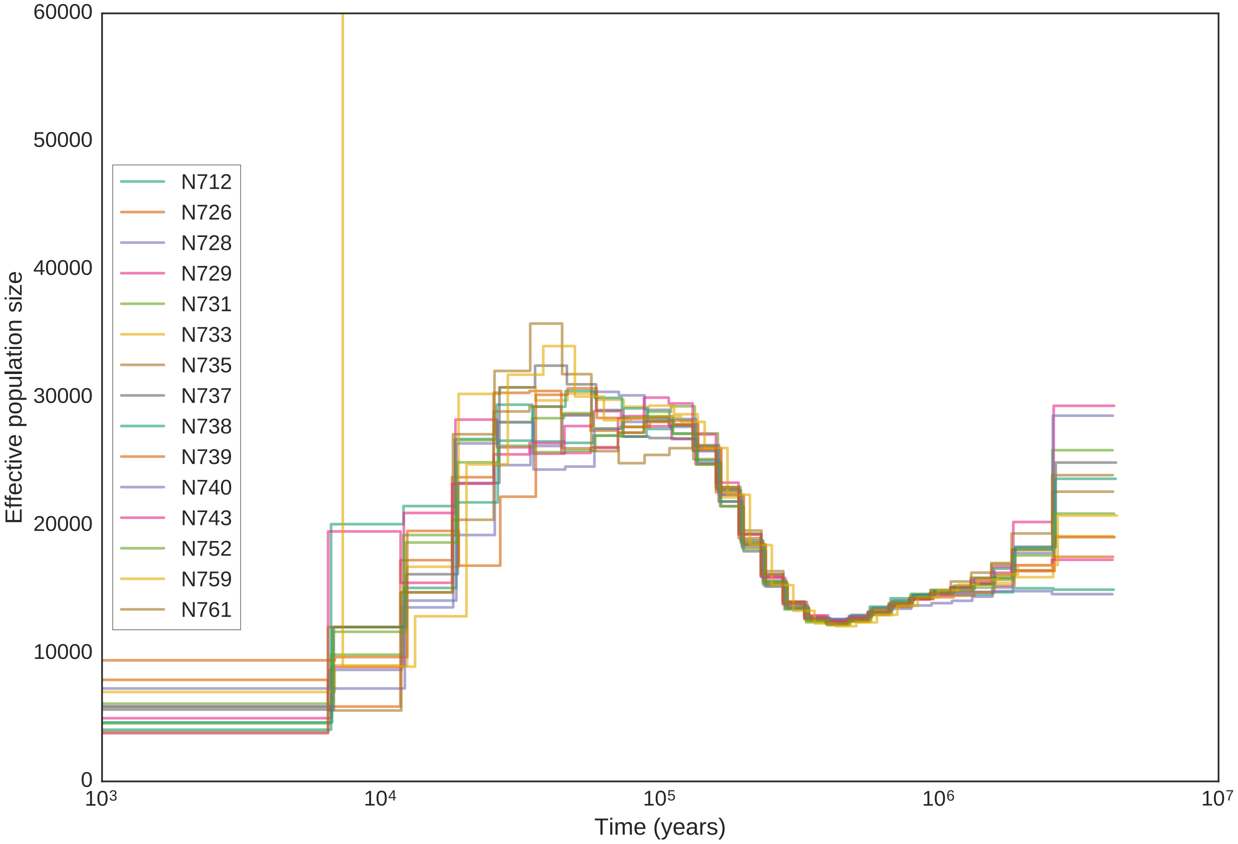

Figure S11: **Historical effective population sizes inferred from CNSW samples using PSMC.** These were scaled using $g = 10$ and $\mu = 7.0 \times 10^{-9}$.

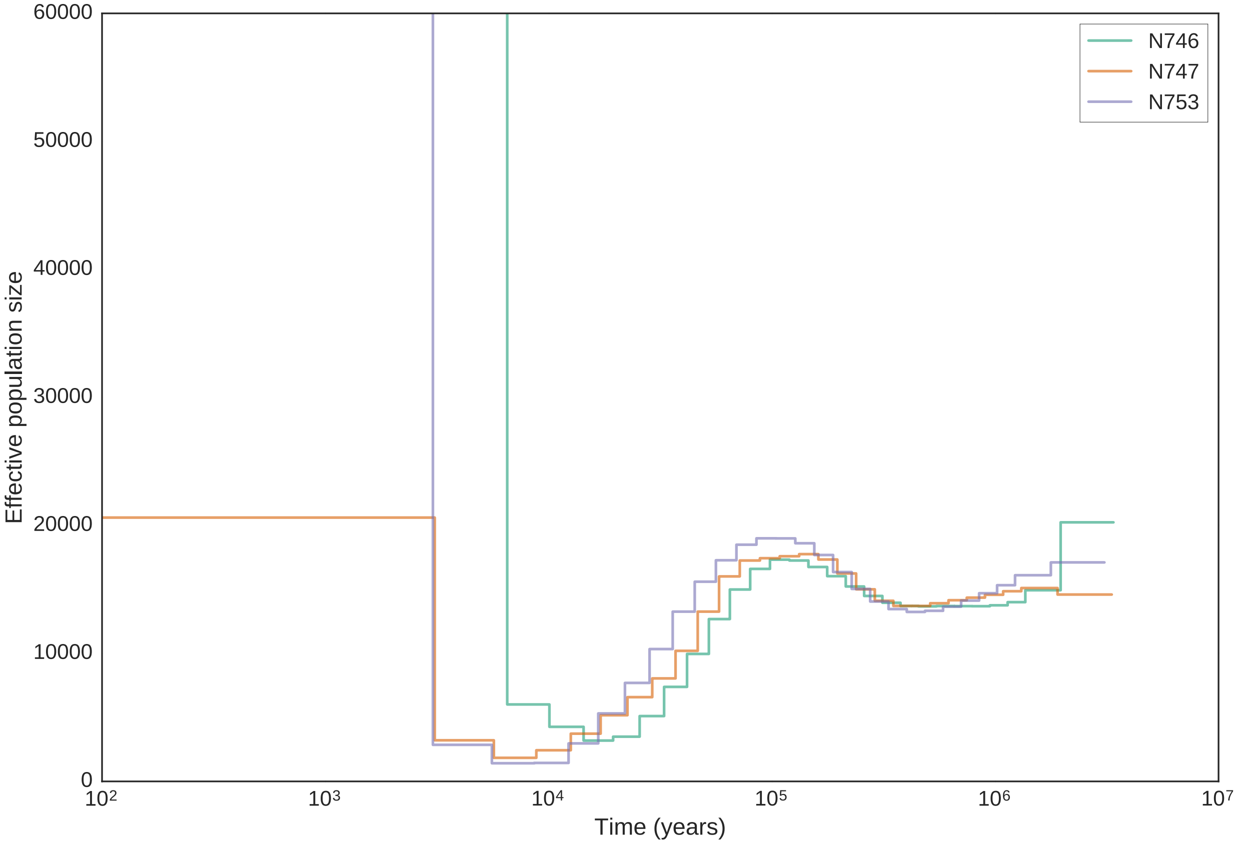

Figure S12: **Historical effective population sizes inferred from central QLD samples using PSMC.** These were scaled using $g = 10$ and $\mu = 7.0\times10^{-9}$.

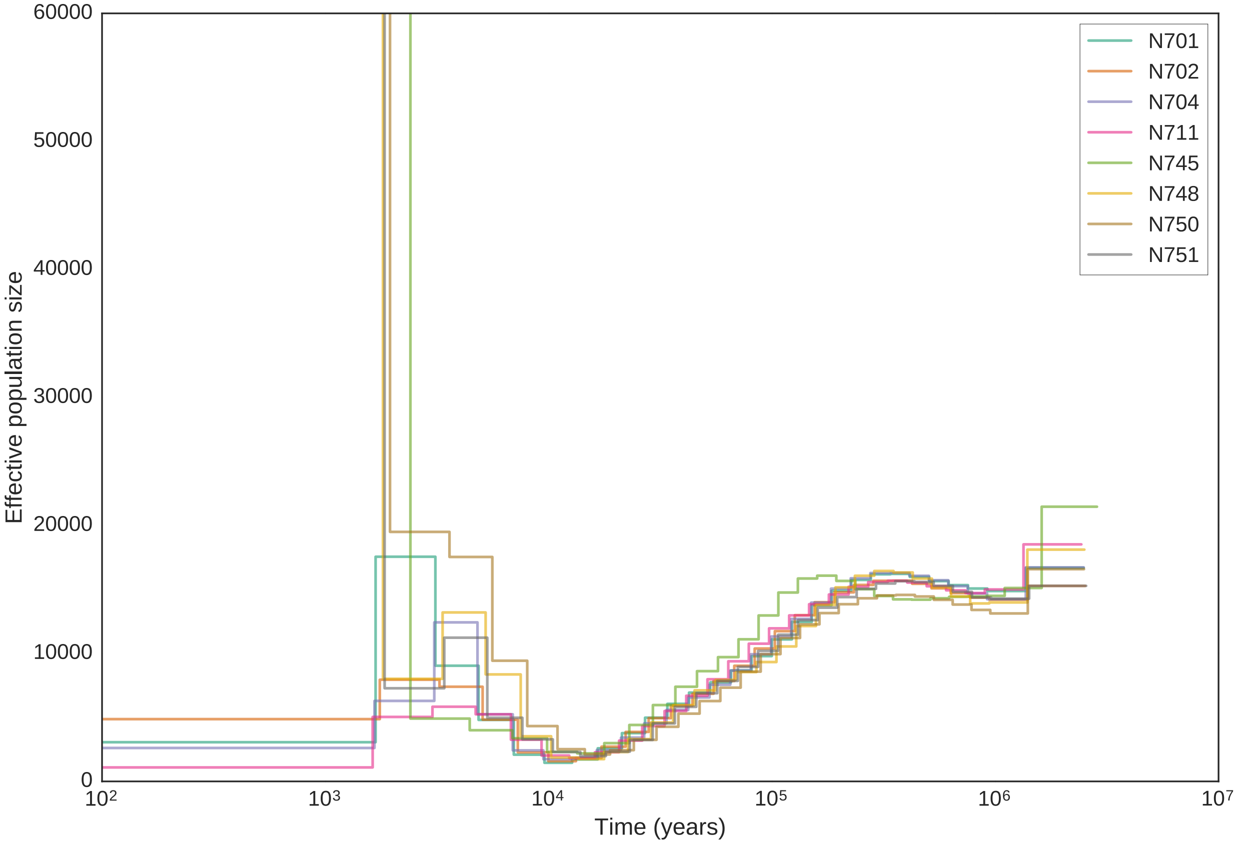

Figure S13: **Historical effective population sizes inferred from north QLD samples using PSMC.** These were scaled using $g = 10$ and $\mu = 7.0 \times 10^{-9}$.

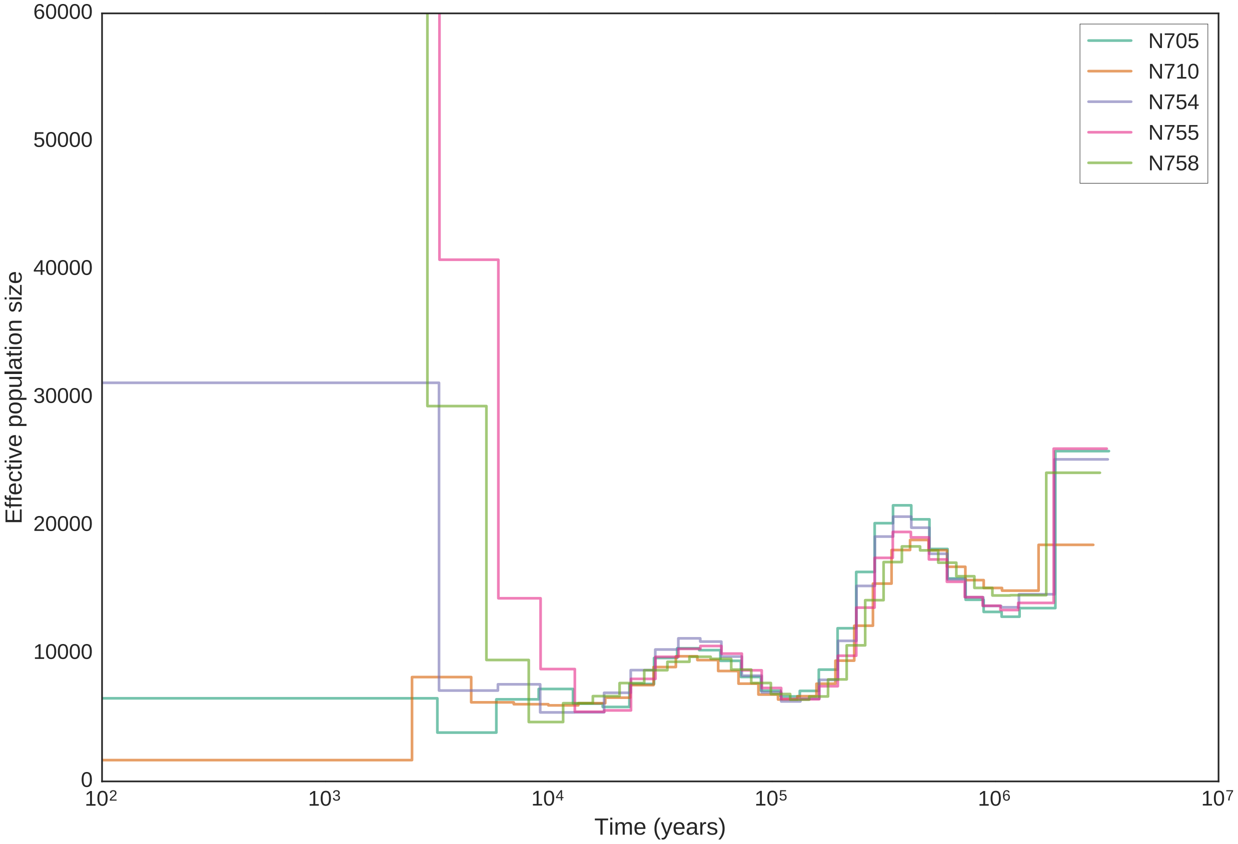

Figure S14: **Historical effective population sizes inferred from Tasmanian samples using PSMC.** These were scaled using $g = 10$ and $\mu = 7.0\times10^{-9}$.

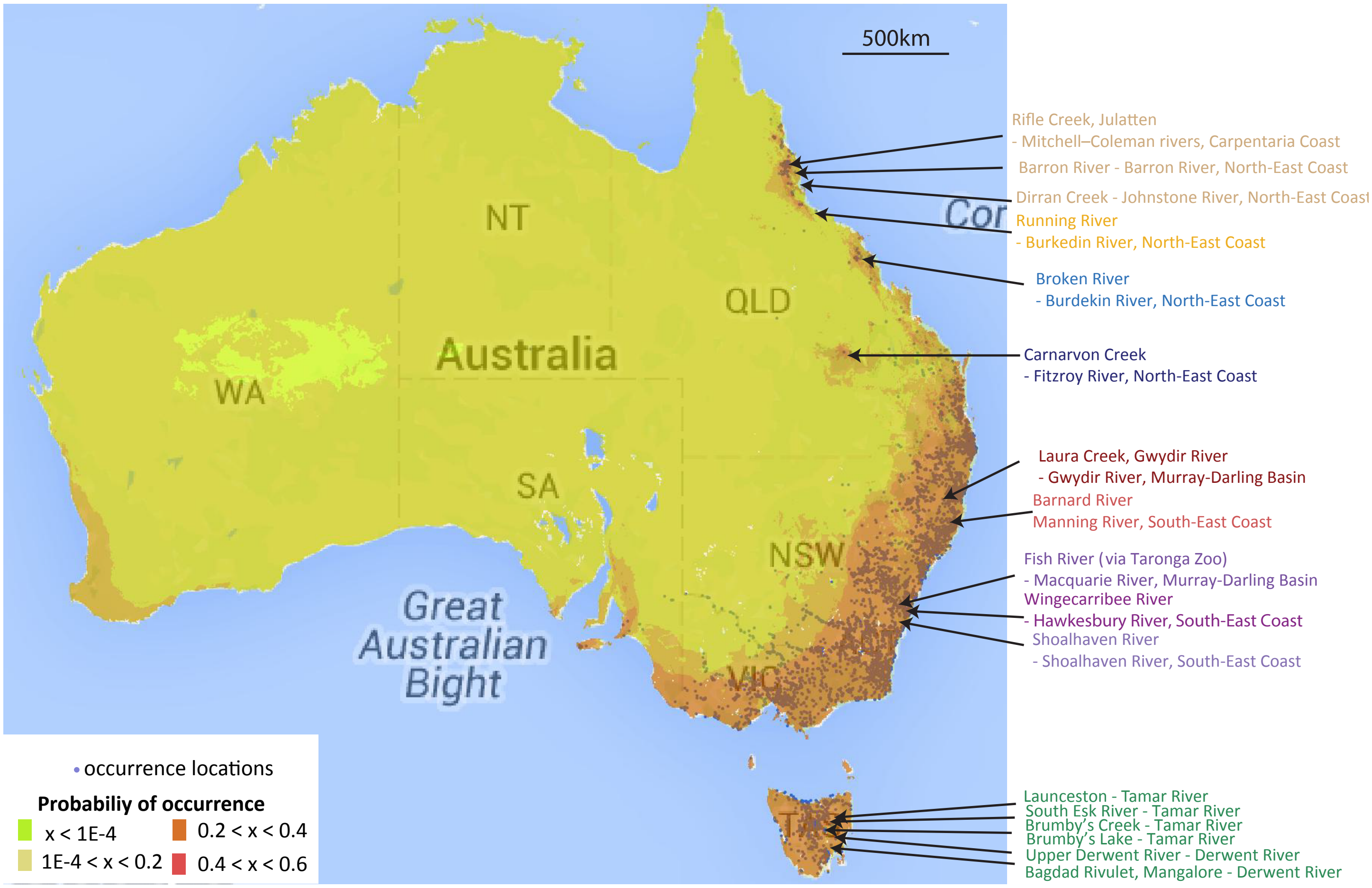

Figure S15: **Observed and expected distribution of platypus.** The arrows indicate our sampling locations, the grey/blue points show occurrence locations and the shading indicates the species habitat predicted using the maximum-entropy approach of Phillips et al. (2006) (see Methods).

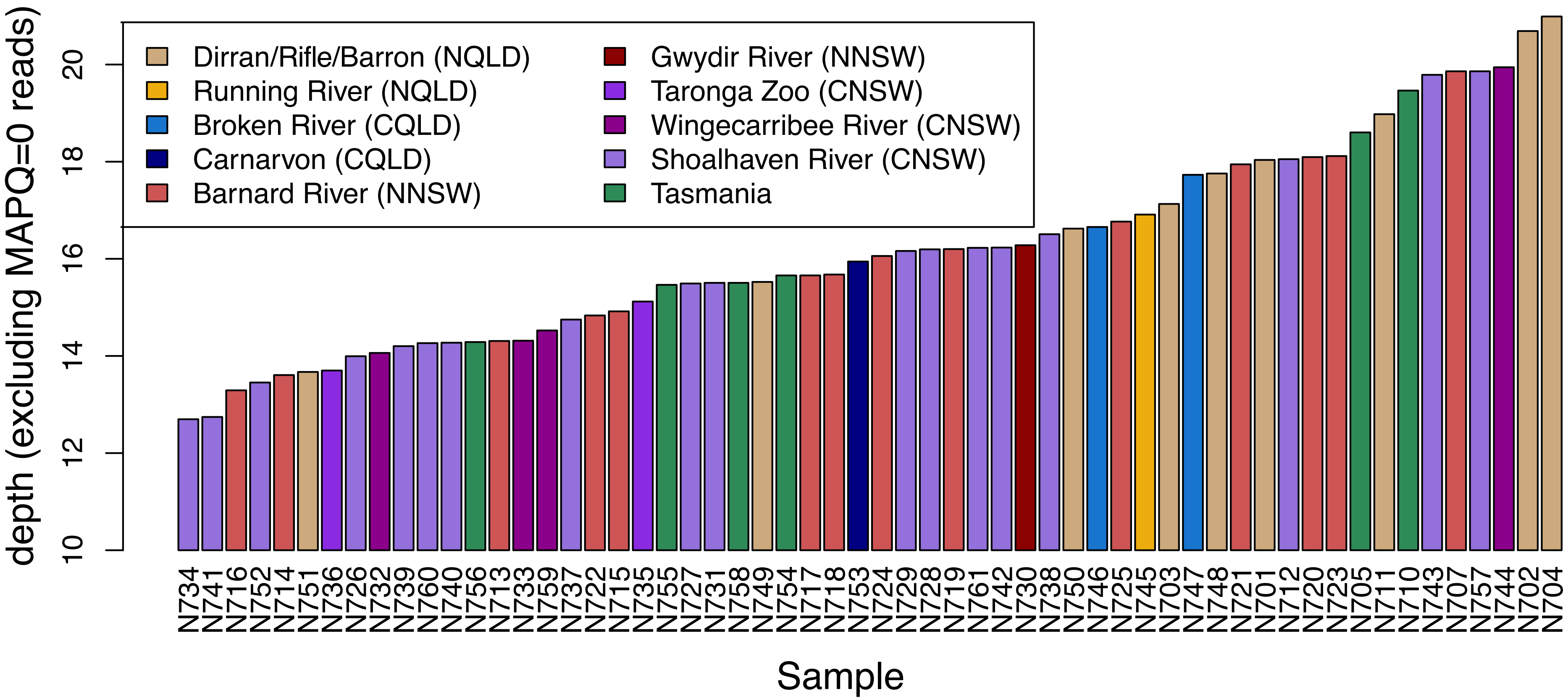

Figure S16: **Average genome-wide coverage per sample.** This is calculated using reads with MAPQ > 0.

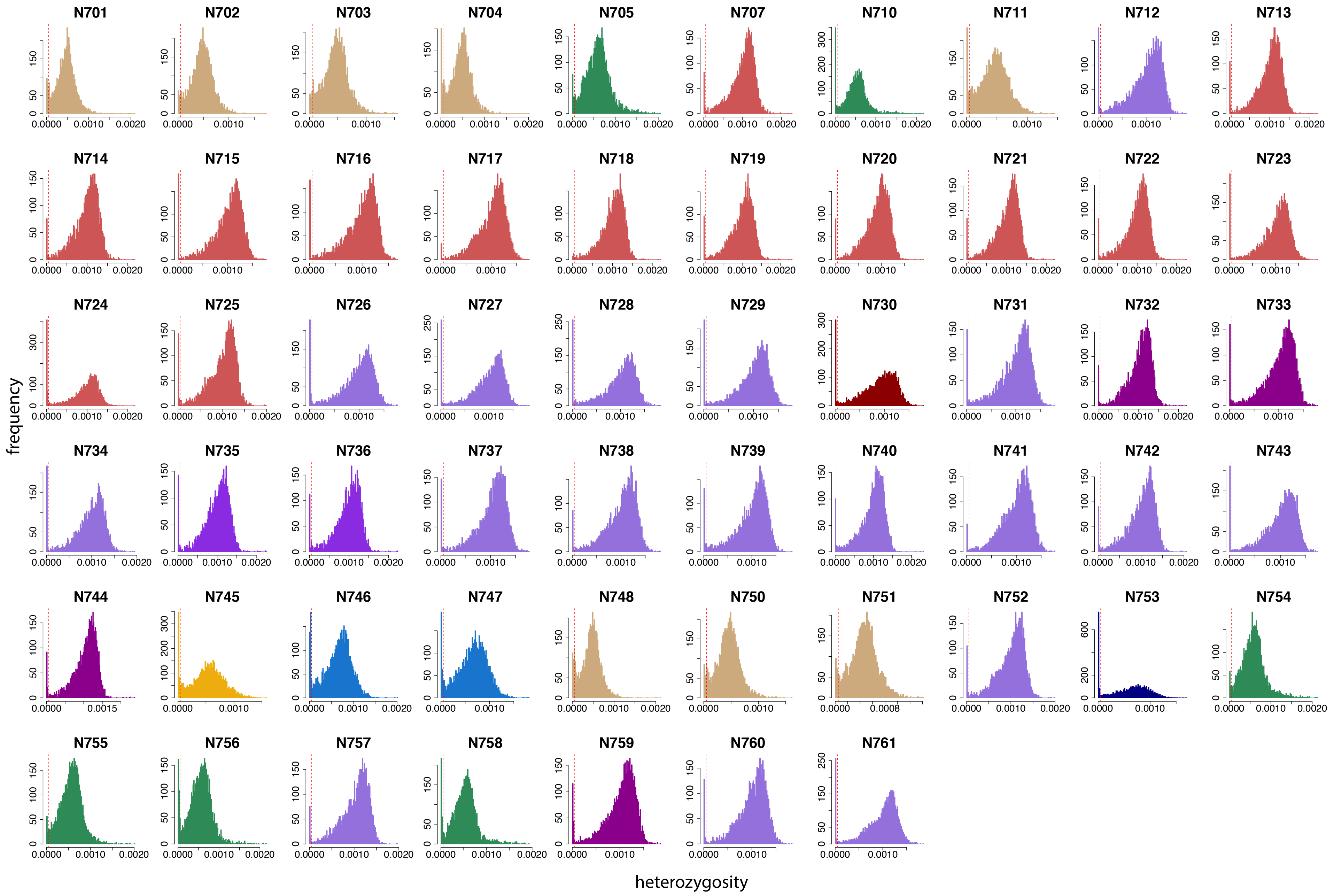

Figure S17: **Histograms of heterozygosity of 1Mb windows with 200kb overlap for each individual.** In this case, the heterozygosity is calculated as the proportion of callable bases that are called as heterozygous. The line indicates the cutoff we used to define windows of homozygosity (5×-5).

S5 Supplementary Tables

Table S1: **Platypus samples sequenced.** The samples in blue are in the unrelated set used for the population genetic analyses (see Methods). The final column indicates how the samples were divided into five groups, and in parentheses, into four more general groups, on the basis of the PCA results. Individuals indicated by N/A were excluded from these groups because there were insufficient samples from that cluster on the PCA shown in Figure 2. NQLD: North Queensland; NNSW: North New South Wales; CNSW: Central New South Wales; TAS: Tasmania.

Sample	Voucher ID	Sex	Sampling Location	Group (larger group)
N701	APlat	M	Dirran Creek	NQLD (NQLD)
N702	BPlat	M		
N703	CPlat	F		
N704	DPlat	F		
N748	PLT035	M		
N749	PLT037	F		
N750	PLT080	F		
N711	PLT010	M	Rifle Creek, Julatten	
N751	PLT033	F	Barron River	
N745	PLT032	F	Running River	N/A (N/A)
N746	PLT068	F	Broken River	N/A (N/A)
N747	PLT069	M		
N753	PLT059	F	Carnarvon Creek	N/A (N/A)
N730	MA428	M	Gwydir River	N/A (NNSW)
N707	GPlat	M	Barnard River	Barnard (NNSW)
N713	P♂2002#1	M		
N714	P♂2002#2	M		
N715	P♂2003	M		
N716	P♂2006	M		
N717	P♂2008#1	M		
N718	P♀2008#1	F		
N719	P♀2008#2	F		
N720	P♂2009#1	M		
N721	P♂2009#2	M		
N722	P♀2009#1	F		
N723	P♂2012#1	M		
N724	P♂2012#2	M		
N725	Glennie	F		
N735	Abby	M	Fish River (Taronga Zoo)	Wingecarribee (CNSW)
N736	Eve	F		
N732	MJ468	F	Wingecarribee River	
N733	Eve	M		
N744	FA624	F		
N759	FJ626	F		
N712	FA556 (SHN42)	F		
N726	FA696	F		
N727	FA531	F		
N728	MA460	M		
N729	MJ380	M		

Continued on next page

Table S1 Continued

Sample	Voucher ID	Sex	Sampling Location	Group (larger group)
N731	MA482	M		
N734	FA513	F		
N737	FA462	F		
N738	MJ402	M		
N739	MJ403	M		
N740	FA667	F		
N741	FA547	F		
N742	FA557	F		
N743	FJ537	F		
N752	MA398	M		
N757	MJ379	M		
N760	FA549	F		
N761	MA393	M		
N705	EPlat	M	Launceston	TAS (TAS)
N710	P29	M	Mangalore	
N754	P150	M	South Esk River	
N755	P114	M	Brumby's Creek	
N756	113	F	Brumby's Lake	
N758	P158	M	Upper Derwent River	

Table S2: **Sequencing coverage and read span by sample.** The coverage is calculated using all mapped reads with MAPQ > 0. The read span is the total distance between the leftmost and rightmost mapping position of the reads in a pair (i.e. twice the read length, plus the distance between the two reads.)

Sample	Tranche	Mean coverage	Mean read span (bp)
N701	1	18.0	224.2
N702	1	20.7	257.1
N703	1	17.1	295.6
N704	1	21.0	250.6
N705	1	18.6	272.3
N707	1	19.9	285.4
N710	2	19.4	361.5
N711	2	19.0	300.8
N712	2	18.1	347.7
N713	3	14.3	367.0
N714	3	13.6	358.8
N715	3	14.9	368.5
N716	3	13.3	371.7
N717	3	15.7	376.9
N718	3	15.7	379.4
N719	3	16.2	375.4
N720	3	18.1	360.8
N721	3	17.9	367.3
N722	3	14.8	367.0
N723	3	18.1	359.5
N724	3	16.1	363.7
N725	3	16.8	368.8
N726	4	14.0	339.5
N727	4	15.5	353.9
N728	4	16.2	352.8
N729	4	16.2	375.8
N730	4	16.3	354.3
N731	4	15.5	363.9
N732	4	14.1	373.8
N733	4	14.3	363.4
N734	4	12.7	394.5
N735	4	15.1	370.2
N736	4	13.7	369.1
N737	4	14.7	351.7
N738	4	16.5	350.1
N739	4	14.2	359.7
N740	4	14.3	349.2
N741	4	12.7	381.5
N742	4	16.2	324.5
N743	4	19.8	376.2
N744	4	19.9	359.0
N745	4	16.9	363.8
N746	4	16.7	343.3
N747	4	17.7	376.7
N748	4	17.8	349.5
N749	4	15.5	355.7
N750	4	16.6	358.9
N751	4	13.7	363.4

Continued on next page

Table S2 Continued

Sample	Tranche	Mean coverage	Mean read span (bp)
N752	4	13.5	382.8
N753	4	15.9	337.6
N754	4	15.7	353.3
N755	4	15.5	352.3
N756	4	14.3	356.1
N757	4	19.9	355.3
N758	4	15.5	352.1
N759	4	14.5	346.9
N760	4	14.3	361.2
N761	4	16.2	366.9

Table S3: **Rate of discordant genotyes between duplicate samples.** The error rate is the proportion of SNPs called that had different genotypes in N703 *versus* N749. IBS: identity-by-state; IBS0: no alleles called in common in both samples i.e. one sample was called 0/0 (homozygous for the reference allele) and the other 1/1 (homozygous for the alternate allele); IBS1: one allele was called in both samples i.e. one sample was 0/1 (heterozygous) and the other was 0/0 or 1/1. Only SNPs not called as missing in either sample were included.

**Error Type**	**Count**	**Error rate per variant**	**Error rate per base**
IBS0	7	1.03×10^{-6}	7.69×10^{-9}
IBS1	14789	2.20×10^{-3}	1.62×10^{-5}
Total	14796	2.20×10^{-3}	1.62×10^{-5}

Table S4: **Related samples identified with KING.**

ID 1	ID 2	Relationship	Kinship	Sampling location
N735	N736	1st degree - siblings	0.257	CNSW
N712	N727	1st degree - siblings	0.258	CNSW
N742	N757	1st degree - siblings	0.261	CNSW
N712	N742	1st degree - parent and child	0.248	CNSW
N752	N757	1st degree - parent and child	0.255	CNSW
N742	N752	1st degree - parent and child	0.254	CNSW
N712	N757	1st degree - parent and child	0.251	CNSW
N702	N749	2nd degree	0.091	NQLD
N755	N756	2nd degree	0.134	TAS
N738	N741	2nd degree	0.097	CNSW
N727	N734	2nd degree	0.110	CNSW
N727	N742	2nd degree	0.116	CNSW
N727	N757	2nd degree	0.131	CNSW
N715	N721	2nd degree	0.140	NNSW
N712	N734	2nd degree	0.129	CNSW
N749	N750	3rd degree	0.071	NQLD
N740	N760	3rd degree	0.057	CNSW
N734	N742	3rd degree	0.071	CNSW
N734	N757	3rd degree	0.058	CNSW
N733	N744	3rd degree	0.069	CNSW
N732	N733	3rd degree	0.081	CNSW
N732	N744	3rd degree	0.071	CNSW
N729	N760	3rd degree	0.085	CNSW
N721	N725	3rd degree	0.073	NNSW
N719	N722	3rd degree	0.047	NNSW
N713	N714	3rd degree	0.055	NNSW

Table S5: **Density of switch errors in the family quartet on ornAna1 *versus* ornAna3.** This indicates a substantial reduction in the number of misassemblies in ornAna3 compared to ornAna1. See Section S1 for a description of how these were assessed.

		**ornAna1**	**ornAna3**
Informative SNPs	maternal	701,983	724,244
	paternal	733,363	737,707
Switches	maternal	371	72
	paternal	319	57
Mean distance between switches	maternal	2,965,626bp	13,386,310bp
	paternal	3,446,827bp	16,909,024bp
Mean informative SNPs per switch	maternal	1,892	10,059
	paternal	2,299	12,942

Table S6: **Library preparation and sequencing.**

Tranche	Date	Samples	Library preparation	Indexes	Sequencer	Read length	Location
1	August 2012	N701-N709[a]	Nextera DNA library preparation kit	Nextera index kit	HiSeq 2000	100bp	WTCHG
2	June 2013	N710-N712	standard Illumina protocol, specifics unknown		HiSeq 2000	100bp	Sydney
3	June 2014	N713-N725	NEBNext; no amplification[b]; 100ng starting material; 1 min fragmentation with Covaris; bead size selected	TruSeq adaptors (barcoded)	HiSeq 2500	150bp	WTCHG
4	February 2015	N726-N761	Wafergen Apollo 324 NGS Library Prep system; 100ng starting DNA; 1 min fragmentation with Covaris	TruSeq adaptors (barcoded)	HiSeq 2500	150bp	WTCHG

[a] Samples N706, N708 and N709 were found to have very uneven coverage (data not shown), thought to be due to low DNA quality, so these were discarded from all analyses.

[b] The libraries for samples N713-N725 were originally prepared using the NEB Ultra DNA Library Prep Kit. However, after the first round of sequencing, some samples were found to have extremely unequal coverage that seemed to be due to differential GC bias during the PCR. Thus, new libraries were produced without the amplification step.
